# Molecular Characterization of *SQUAMOSA PROMOTER BINDING PROTEIN-LIKE (SPL)* Gene Family in *Betula luminifera*

**DOI:** 10.3389/fpls.2018.00608

**Published:** 2018-05-04

**Authors:** Xiu-Yun Li, Er-Pei Lin, Hua-Hong Huang, Ming-Yue Niu, Zai-Kang Tong, Jun-Hong Zhang

**Affiliations:** The State Key Laboratory of Subtropical Silviculture, Institute of Biotechnology, College of Forestry and Biotechnology, Zhejiang A&F University, Lin'an, China

**Keywords:** *Betula luminifera*, *SPL* gene family, miR156, expression patterns, vegetative phase change, DELLA proteins

## Abstract

As a major family of plant-specific transcription factors, *SQUAMOSA PROMOTER BINDING PROTEIN-LIKE* (*SPL*) genes play vital regulatory roles in plant growth, development and stress responses. In this study, 18 *SPL* genes were identified and cloned from *Betula luminifera*. Two zinc finger-like structures and a nuclear location signal (NLS) segments were existed in the SBP domains of all BlSPLs. Phylogenetic analysis showed that these genes were clustered into nine groups (group I-IX). The intron/exon structure and motif composition were highly conserved within the same group. 12 of the 18 *BlSPL*s were experimentally verified as the targets of miR156, and two cleavage sites were detected in these miR156-targeted *BlSPL* genes. Many putative cis-elements, associated with light, stresses and phytohormones response, were identified in the promoter regions of *BlSPL*s, suggesting that *BlSPL* genes are probably involved in important physiological processes and developmental events. Tissue-specific expression analysis showed that miR156-targeted *BlSPL*s exhibited a more differential expression pattern, while most miR156-nontargeted *BlSPL*s tended to be constitutively expressed, suggesting the distinct roles of miR156-targeted and nontargeted *BlSPL*s in development and growth of *B. luminifera*. Further expression analysis revealed that miR156-targeted *BlSPL*s were dramatically up-regulated with age, whereas mature BlmiR156 level was apparently declined with age, indicating that miR156/*SPL* module plays important roles in vegetative phase change of *B. luminifera*. Moreover, yeast two-hybrid assay indicated that several miR156-targeted and nontargeted BlSPLs could interact with two DELLA proteins (BlRGA and BlRGL), which suggests that certain *BlSPL*s take part in the GA regulated processes through protein interaction with DELLA proteins. All these results provide an important basis for further exploring the biological functions of *BlSPL*s in *B. luminifera*.

## Introduction

*SQUAMOSA PROMOTER BINDING PROTEIN-LIKE* (*SPL*) proteins represent a specific transcription factor family in plant. The common characteristic of SPL proteins is the highly conserved DNA-binding domain termed the SBP domain (Yamasaki et al., 2004; Birkenbihl et al., 2005). This domain is comprised of approximately 76 amino acid residues containing two zinc-binding sites, and involved in DNA-binding and nuclear localization (Yamasaki et al., 2004). Due to their ability of binding the promoter region of the floral meristem identity gene *SQUAMOSA, SPL* genes were firstly identified in *Antirrhinum majus* (Klein et al., 1996). Lately, *SPL* genes are identified in all green plant lineages, including green alga, moss, lycophyte, gymnosperm and angiosperm (Riese et al., 2007; Guo et al., 2008; Yang et al., 2008; Li and Lu, 2014; Zhang et al., 2016). And, *SPL* genes exist as a multigene family in plants. For example, 16 *SPL* genes are found in *Arabidopsis thaliana* (Cardon et al., 1999), 19 in rice (Xie et al., 2006; Yang et al., 2008), and 28 in *P. trichocarpa* (Li and Lu, 2014). Many of *SPL* genes are important regulators for several diverse plant developmental processes including vegetative phase change (Wang et al., 2009), plant architecture (Jiao et al., 2010; Gou et al., 2017), anthocyanin biosynthesis (Gou et al., 2011), gibberellin (GA) biosynthesis and signaling (Zhang et al., 2007; Yu et al., 2012), somatic embryogenesis (Wang et al., 2017), and in plant response to stresses (Cui et al., 2014).

MiRNAs are a class of noncoding 20–24 nt small RNAs that play important roles in posttranscriptional gene regulation by targeting mRNAs for cleavage or repressing translation in plant (Zhang et al., 2006; Chen, 2009). Many *SPL* genes are targeted by miR156, which is one of the conserved plant miRNA families. The miR156/SPL module has shown their critical regulatory roles in many development processes (Jiao et al., 2010; Lal et al., 2011; Wang and Wang, 2015). In monocots, the miR156/SPL module has been proposed as a tool to genetically enhance the agronomic traits to maximize the yield in corps (Jiao et al., 2010; Wang and Wang, 2015). For instance, higher expression of *OsSPL14*, which is regulated by *OsmiR156 in vivo*, promotes panicle branching in the reproductive stage and increases grain yield in rice (Jiao et al., 2010; Miura et al., 2010). Overexpression of miR156 induced aerial bud formation in switchgrass, while down-regulation of *SPL4*, one of the miR156 targets, promoted aerial bud formation and increased basal buds, which are closely related to the biomass productivity (Gou et al., 2017). In *Arabidopsis*, the 3′UTR of *AtSPL3* mRNA contains complementary sequences of miR156, and its expression is mediated by miR156 through translation inhibition and transcript cleavage, which defines an important regulatory module of vegetative phase change and floral transition in *Arabidopsis* (Gandikota et al., 2007; Wang et al., 2009; Yamaguchi et al., 2009). Overexpression of miR156 in transgenic *Populus x canadensis* decreased the expression of miR156-targeted *SPL* genes resulting in the severely prolonged juvenile phase, suggesting an evolutionarily conserved role of miR156/SPL module in vegetative phase change of annual herbaceous plants and perennial trees (Wang et al., 2011).

DELLA proteins represent a subgroup of the GRAS family of plant-specific transcription factors, and act as the key negative regulator of GA signaling (Sun, 2010; Locascio et al., 2013). To establish protein-protein interaction with diverse classes of regulatory proteins is the major way to exert function for DELLA proteins (Locascio et al., 2013). By the protein interactions, DELLAs respectively inhibit the DNA-binding ability of transcription factors or the activity of transcriptional regulators, which are important in regulation of various plant development processes (Davière et al., 2014; Rombolá-Caldentey et al., 2014; Huang et al., 2015; Yu et al., 2016). SPL proteins have been revealed as the transcriptional regulators that interact with DELLA proteins. In *Arabidopsis*, DELLA proteins directly bind to miR156-targeted SPL transcription factors, which disturbs SPL transcriptional activity and consequently delays floral transition (Yu et al., 2012). Therefore, the interaction between SPL and DELLA is thought to be critical for GA pathway in floral transition.

*Betula luminifera* H. Winkler, a broadleaf tree species, is widely distributed in southern China. Due to its desirable wood properties and fast-growth rate, *B. luminifera* has been widely grown for timber, being used in high quality furniture, wood veneers and solid wood flooring. In addition to its good economic traits, *B. luminifera* has a relatively short juvenile period, and many germplasms start flowering within 18 months. Such short vegetative growth phase undoubtedly accelerate the breeding progress of this timber tree, which makes it possible to take *B. luminifera* as a model for tree breeding.

Although the important roles of *SPL* genes have been illustrated in model plant, the function of *SPL* genes in *B. luminifera* is largely unknown. In the current study, the intron/exon structure, phylogeny, conserved motif, miRNA-mediated posttranscriptional regulation and *cis*-elements in promoter regions were systematically investigated for *SPL* genes in *B. luminifera*. Meanwhile, expression patterns of *BlSPL*s and mature BlmiR156 in various tissues and organs, and in plants with different ages, were investigated to study the possible roles of *BlSPL* genes in development, especially in vegetative phase change, of *B. luminifera*. Moreover, the interactions between BlSPLs and two DELLA proteins were also examined through yeast two-hybrid to study their involvements in the GA regulated biological processes. Our findings provide a solid foundation for future research to elucidate the functions of the *SPL* genes in *B. luminifera*, and will surely promote the utilization of these genes.

## Materials and methods

### Plant materials

Plant samples from *Betula luminifera* (clone 1V25-2), which grown in the Tree Germplasm Garden of Zhejiang A&F University, were used for gene cloning and expression analysis. For the gene cloning, stems, leaves, and inflorescences were collected from 3-year-old plants in April, and then stored in liquid nitrogen until use. For expression analysis in different tissues and organs, buds, leaves, male inflorescences, female inflorescences, stems, root, phloem, xylem, and seeds were collected from 3-year-old plants between early March and Late May. For expression analysis in seedling/plants with different ages, the seedlings germinated from self-pollinated seeds of clone 1V25-2, and the 3-, 5- month-old, 1.5- and 4-year-old trees of clone 1V25-2 were used. The 5-, 10-, and 21-day-old seedlings being excised roots, and the 2-3 cm newly-expanded leaves from the shoot apex of trees were collected and stored in liquid nitrogen until use.

### Identification of *BLSPL* genes

The amino acid sequences of AtSPLs were retrieved from the Arabidopsis Information Resource database (TAIR version 10, http://www.arabidopsis.org/), and the sequences of *Populus* PtSPLs were obtained from the previous study (Li and Lu, 2014). To identify the genomic sequences (contigs) harboring *BlSPL* genes, the AtSPL and PtSPL protein sequences were used for TBLASTN search (Altschul et al., 1997) against the current assembly of the *B. luminifera* genome (Huang et al., unpublished data 2017) with the parameter “-evalue 1e-5”. Then, the candidate contigs identified by TBLASTN (Supplementary Table S1) were used to predict the gene models of *BlSPL*s using the FGENESH online tool with the genome-specific gene-finding parameters of *A*. *thaliana* and *P. trichocarpa* (Softberry, http://www.softberry.com/berry.phtml; Solovyev et al., 2006). All the nonredundant gene models were submitted to Interpro (http://www.ebi.ac.uk/interpro) to confirm the presence of the SBP domain. Based on the predicted models, all the *BlSPL* genes were cloned and sequenced, and the predicted gene models were then further manually corrected and verified by alignment with the cloned sequences. The predicted and corrected gene models were listed in the Supplementary Table S2. All the sequences of *BlSPL* genes were deposited in Genbank (https://www.ncbi.nlm.nih.gov/genbank/) and the accession numbers were listed in Table 1.

**Table 1:** Gene feature and classification of *SPLs* in *Betula luminifera*.

**Gene name**	**Accession No**.	**Gene length(bp)**	**CDS(bp)**	**Peptide (aa)**	**Mw(Da)**	**p*I***	**No. intron**	**Group**	**miR156 target**
*BlSPL1*	AGC92796	1894	1170	389	42343.83	8.43	2	VII	√
*BlSPL2*	KY548818	1316	930	309	34392.50	8.98	2	III	
*BlSPL3*	KY548819	720	405	134	15441.11	6.39	1	VI	√
*BlSPL4*	KY548820	4170	3009	1002	110596.28	6.08	9	II	
*BlSPL5*	KY548821	4234	3249	1082	119424.52	7.45	9	II	
*BlSPL6*	KY548822	2129	1443	480	53214.71	7.34	3	V	√
*BlSPL7*	KY548823	2251	1407	468	51527.92	8.23	3	V	√
*BlSPL8*	KY548824	1838	1134	377	40244.57	9.34	2	VIII	√
*BlSPL9*	KY548825	1613	1173	390	42739.94	8.28	2	VII	√
*BlSPL10*	KY548826	3181	2403	800	88946.27	6.27	9	I	
*BlSPL11*	KY548827	2094	1389	462	50910.04	8.29	2	IV	√
*BlSPL12*	KY548828	3263	2949	982	108710.48	6.97	9	II	
*BlSPL13*	KY548829	1936	1503	500	54689.22	8.06	2	IV	√
*BlSPL14*	KY548830	1141	630	209	24307.43	5.89	1	VI	
*BlSPL15*	KY548831	1015	648	215	24331.98	9.08	1	VI	√
*BlSPL16*	KY548832	1743	1245	414	45549.83	7.77	3	IX	√
*BlSPL17*	KY548833	1457	528	175	19957.82	9.04	1	VI	√
*BlSPL18*	KY548834	1905	975	324	35229.92	9.01	2	VII	√

### Phylogenetic analysis

Multiple sequences alignment of SPL proteins was performed using AlignX to obtain SBP domain sequences. Phylogenetic analysis was carried out by the neighbor-joining (NJ) method in MEGA 7.0 with 1,000 bootstrap replicates (Kumar et al., 2016).

### Bioinformatic analyses of *BLSPL* genes

The theoretical isoelectric point (p*I*) and molecular weight (Mw) of BlSPLs were calculated using the Compute p*I*/Mw tool on the ExPASy server (http://web.expasy.org/compute_pi/). Intron/exon structures were displayed by mapping the cDNA sequences of *BlSPL*s to the corresponding genomic sequences using the Gene Structure Display Sever 2 (Hu et al., 2015; http://gsds.cbi.pku.edu.cn/). Conserved domains of BlSPLs were identified using Pfam (http://pfam.sanger.ac.uk) and the Conserved Domain Database (CDD, http://www.ncbi.nlm.nih.gov/Structure/cdd/wrpsb.cgi) with the expected E-value threshold of 0.01 and the maximum hits of 500 amino acids (Zhang et al., 2013). Sequence logos were generated by using WebLogo (http://weblogo.berkeley.edu/). Sequence alignment of SBP domains, ANK domains and miR156 complementary sequences was carried out using Vector NTI 11.0 (Invitrogen). Conserved motifs were determined using MEME version 4.11.4 (Bailey and Elkan, 1994) (http://meme-suite.org/tools/meme) with the parameters as previous study (Li and Lu, 2014). MiR156-targeted *BlSPL*s were predicted using psRNATarget tool (http://plantgrn.noble.org/psRNATarget/; Dai and Zhao, 2011).

### Cis-elements analysis

Putative cis-elements in the promoter regions (1,500 bp upstream sequences of the start codon) of *BlSPL*s were annotated using PlantCARE (http://bioinformatics.psb.ugent.be/webtools/plantcare/html/) database (Lescot et al., 2002). The motifs putatively involved in plant growth and development, phytohormone responses, and stress responses are summarized.

### RNA isolation

Total RNA was extracted from tissues or organs of *B. luminifera* using the PureLink Plant RNA Reagent (Ambion) according to the manufactuer's instructions. Agarose gel electrophoresis and nanodrop 2000 spectrophotometer (Thermo Scientific) were used to evaluate the quality and quantity of total RNA, and genomic DNA was eliminated by treatment of RNase-free DNase (Promega).

### Molecular cloning of *BLSPL*s

According to SMARTer RACE cDNA Amplification Kit (ClonTech), the full length of *BlSPL*s were obtained by RACE method using cDNA of *Betula luminifera* as template by PCR, and using the gene specific forward and reverse primers listed in Supplementary Table S3. The complete coding regions of *BlSPL*s were amplified and verified by PCR using the specific primers listed in Supplementary Table S3. PCR products were purified, cloned into pMD-19 T vector (Takara), and then were sent to sequencing (Genscript, Nanjing).

### Experimental validation of cleavage of miR156-targeted *BLSPL*s

To identify the cleavage sites of miR156-targeted *BlSPL*s, the modified RNA ligase-mediated (RLM) rapid amplification of 5' cDNAs method (5' RLM-RACE) was performed using the First Choice RLM-RACE Kit (Invitrogen) as described previously (Guo et al., 2005). Nested gene specific primers were designed according to the predicted cleavage sites and are listed in Supplementary Table S4. The 5'-RACE products were purified and cloned into pEAST-T1 vector (TransGen Biotech, Beijing). At least eight clones were taken and confirmed by Sanger sequencing.

### Expression analysis of *BLSPL* and *DELLA* genes

The cDNA was synthesized according to the manufacturer's instructions of PrimeScript RT reagent Kit (Takara). The quantitative reverse transcription-PCR (qRT-PCR) was performed on the CFX96 thermocycler (Bio-Rad) using SYBR Green qPCR Master Mix (Takara). The program used for qRT-PCR is as follows: pre-denaturation at 95°C for 30 s, 40 cycles of amplification at 95°C for 5 s, annealing at 60°C for 20 s and 72°C for 15 s. Elongation factor 1-alpha gene (*EF1*α) (Genbank accession no. KM586061) was used as a reference gene as described previously (Liu et al., 2016). Melting curve was used to evaluate amplification specificity. The relative expression levels of *BlSPL* and *DELLA* genes were analyzed using the 2^−ΔΔCq^ method (Schmittgen and Livak, 2008). Normalization of gene expression data from three biological replicates was performed as described (Willems et al., 2008). All the primers used for qRT-PCR are listed in Supplementary Table S5.

### Expression analysis of BlmiR156

Expression of BlmiR156 was analyzed using specific reverse transcription primers as the method described in the previous studies (Varkonyi-Gasic et al., 2007; Li et al., 2013). Briefly, 200 ng total RNA treated by DNase was used for first-strand cDNA synthesis using PrimeScript 1st Strand cDNA Synthesis Kit (TaKaRa). A reaction volume of 10 μL was set up according to the manufacturer's instructions, using 0.5 μL miR156 reverse transcription primer (10 mM) and 0.1 μL 5.8S rRNA reverse primer (10 mM) instead of Poly dT and random hexamer primers. The qRT-PCR was performed as described above. The 5.8S rRNA (GenBank accession no. KT308944) was used as a reference gene in the qRT-PCR. The primers used in the expression analysis of BlmiR156 are listed in Supplementary Table S5.

### Yeast two hybrid assay

The yeast strains and media used for the yeast two-hybrid experiment were provided in the Matchmaker Golden Yeast Two-Hybrid System (Clontech). The open reading frames (ORF) of *BlSPL* genes, *BlRGA* (Genbank accession no. MF149049) and *BlRGL* (Genbank accession no. MF149050) were amplified and firstly cloned into pCR™8/GW/TOPO vector (Invitrogen). Then, these ORFs were cloned into pDEST-GBKT7 (GAL4 DNA binding domain, BD) or pDEST-GADT7 (GAL4 activation domain, AD; Rossignol et al., 2007) through LR recombination reaction (Invitrogen). The BD fused constructs and AD fused constructs were transformed into Y2HGold cells and Y187 cells by the lithium acetate-mediated method, respectively. The yeast two-hybrid assay was performed by yeast mating according to the user manual. The BD fused constructs and AD fused constructs were tested for their autoactivation and toxicity, and those constructs without autoactivation and toxicity in yeast cells were used for yeast two-hybrid assay (Supplementary Figure S1). To measure the transcription activation activity, β-Galactosidase activity was assayed using o-nitrophenyl β-D-galactopyranoside (ONPG) as described in Yeast Protocol Handbook (Clontech). The primers used for ORF amplification are listed in Supplementary Table S6.

## Results

### Identification, molecular cloning and gene feature analysis of *BLSPL*s

In order to identify *SPL* genes in *B*. *luminifera*, all SPL proteins of *Arabidopsis* and *Populus* were used to query against the draft genome (Huang et al, unpublished data, 2017) using TBLASTN algorithm (Altschul et al., 1997). The genomic sequences identified by TBLASTN were then used to predict gene models by using the FGENESH online tool. As shown in the Supplementary Table S2, 18 gene models containing complete SBP-domain were obtained. These predicted gene models were further verified and corrected through molecular cloning and sequencing. The corrected gene models indicated the existence of 18 *BlSPL* genes in genome of *B. luminifera*, and were named *BlSPL1* to *BlSPL18* (Table 1, Supplementary Table S2).

Further analysis of the experimentally validated cDNA sequence of *BlSPL*s showed that the lengths of *BlSPL* cDNAs varied between 720 and 4,234 bp, and the deduced protein lengths from 134 to 1,082 amino acids (Table 1). The theoretical p*I* of deduced BlSPL proteins ranged from 5.89 to 9.34, and the molecular weight (Mw) varied from 15.4 to 119.4 kDa (Table 1). In addition, the number of introns varied between 1 and 9 in *BlSPL* genes (Table 1, **Figure 3**), which is similar with the number of *AtSPL* and *PtSPL* genes. These results revealed the diversity of *SPL* genes features in *B. luminifera*.

### Analyses of conserved domain, phylogeny, and intron/exon structure

**Figure 1:**
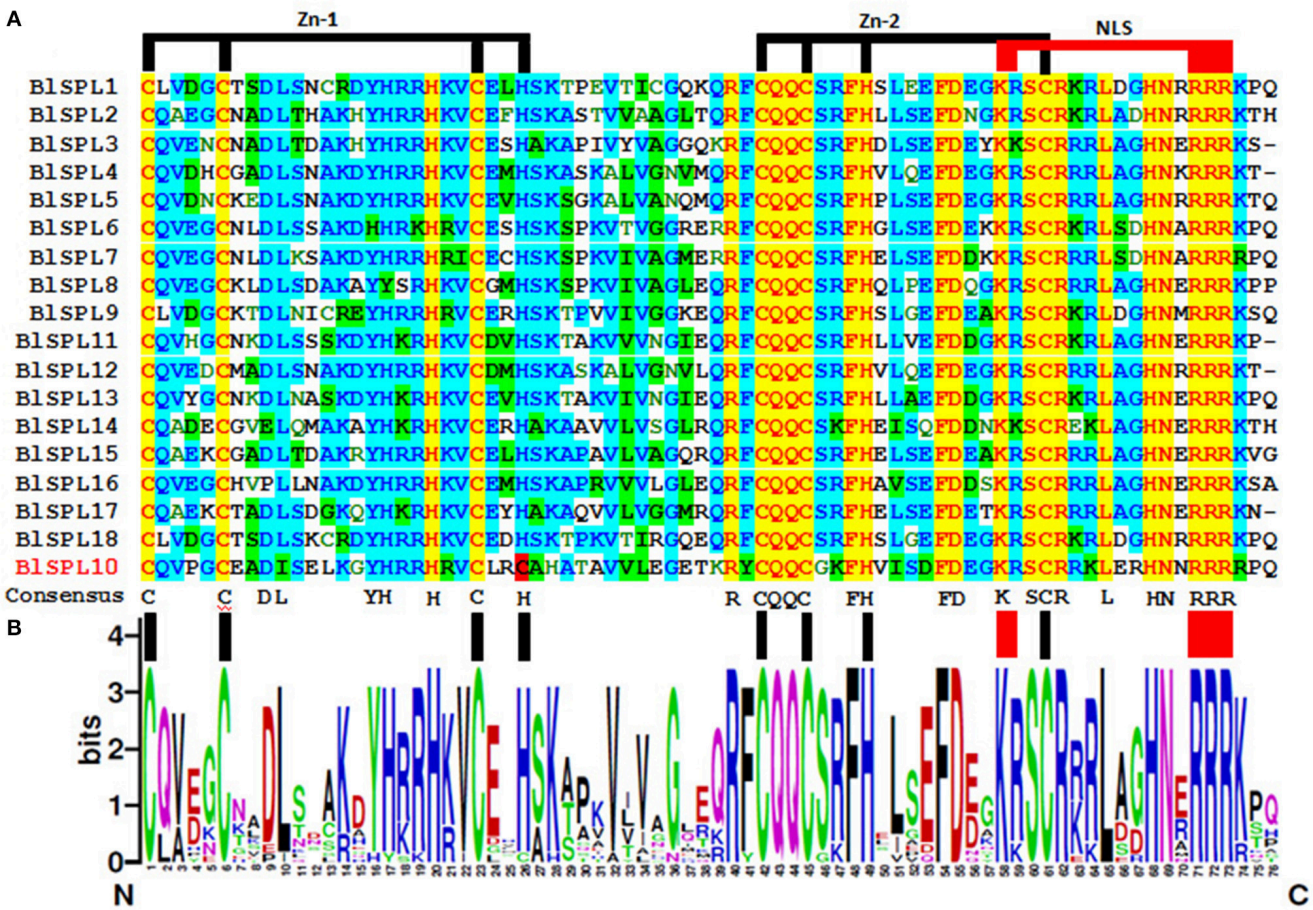
SBP-domain alignment of BlSPLs. **(A)** Multiple alignment of the SBP domains of the *B. luminifera* SBP-box proteins was obtained with the Vector NTI Advanced 11 software. The two conserved zinc finger structures (Zn-1, Zn-2) and NLS were indicated. **(B)** Sequence logo of the SBP domain of BlSPLs. The overall height of each stack represents the degree of conservation at this position, while the height of the letters within each stack indicates the relative frequency of the corresponding amino acids.

The detailed domain structures of BlSPLs were analyzed by multiple sequence alignment. As the result, SBP domain was the only one conserved domain found to be shared by all BlSPLs (Figure 1A). High similarity of the SBP domains, especially several extremely conserved positions, was observed in all BlSPLs (Figure 1B). All of the SBP domains in BlSPLs shared two zinc finger-like structures (Zn-1, Zn-2). The Zn-1 motif was C3H (CCCH) type in all the SPL proteins except BlSPL10, in which the His residue was replaced by Cys residue (Figure 1A). Unlike Zn-1, the signature sequence (C2HC) of Zn-2 is highly conserved in all BlSPLs. Except for the zinc finger-like structure, all the BlSPLs contain a conserved nuclear location signal (NLS) in the C-terminus of SBP domains, which was partly overlapped with the Zn-2 motif (Figure 1A). These structure features of SBP domains were also observed in other plant SPLs (Yang et al., 2008; Li et al., 2013; Li and Lu, 2014; Zhang et al., 2016), indicating the highly conservation of SBP domain in plants. Moreover, three BlSPLs (BlSPL4, BlSPL5, and BlSPL12) contain an ANK or Ank-2 domain with four or three ankyrin repeats (Supplementary Figure S2), which were less conserved than SBP domain and believed to be associated with protein-protein interaction (Michaely and Bennett, 1992).

**Figure 2:**
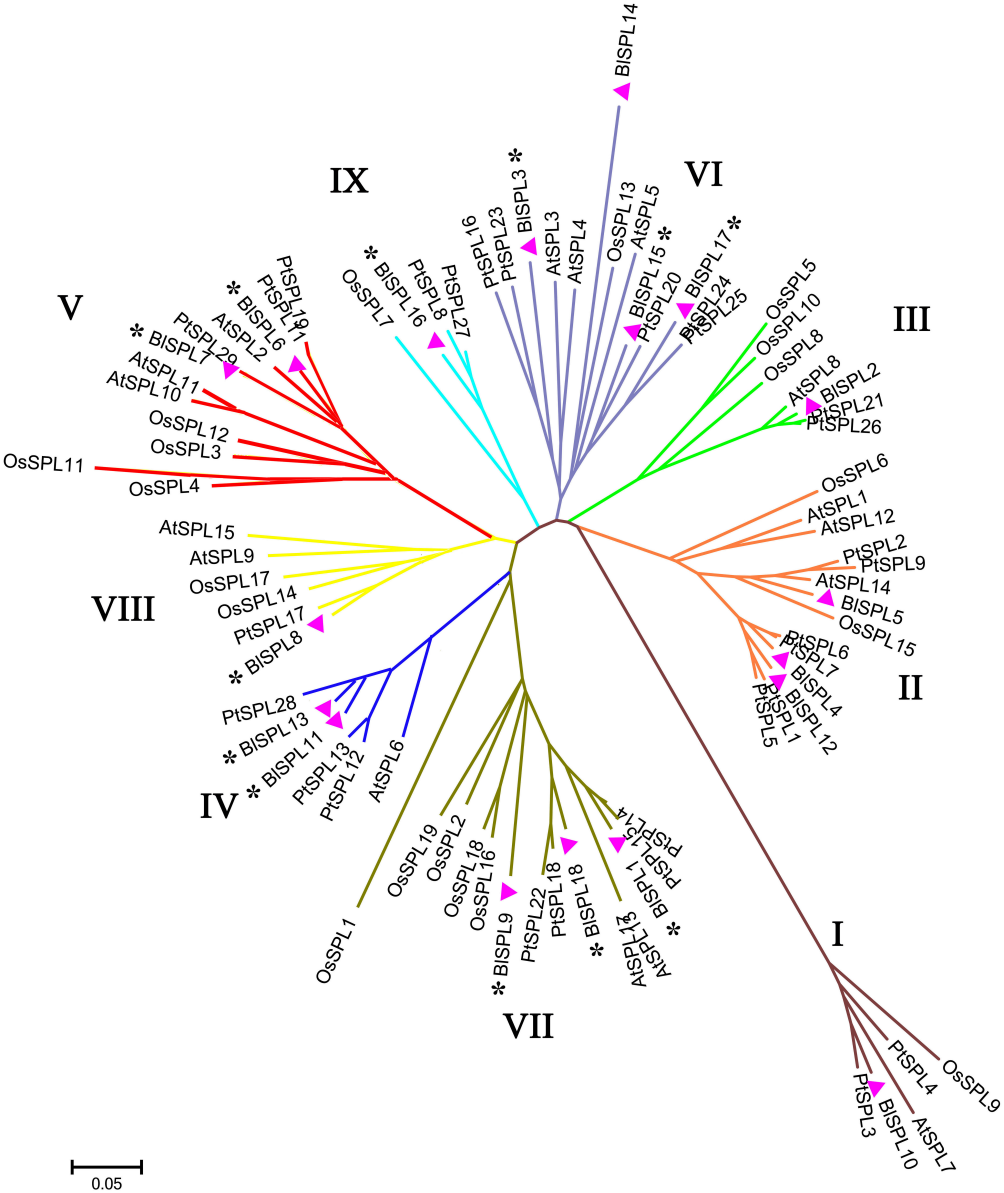
Phylogenetic analysis of *B. luminifera* and other plant SPLs. Phylogenetic tree was constructed with SBP domain protein sequences from *B. luminifera* (BlSPL), rice (OsSPL), *P. trichocarpa* (PtSPL), and *Arabidopsis* (AtSPL). SPL genes from *B. luminifera* that contain complementary sequences to mature BlmiR156 are marked with asterisks. SBP domain sequences of all genes used for phylogenetic tree construction, as well as the accession numbers or locus IDs and data sources of SPL genes from different species are listed in Supplementary Tables S7, S8.

In order to investigate the evolutionary relationship of *BlSPLs* with *SPL* genes from other plants, 81 SPL sequences were collected to construct an unrooted phylogenetic tree using neighbor-joining (NJ) method in MEGA7.0. These 81 SPLs were selected from four plant species, including 18 from *B. luminifera*, 16 from *Arabidopsis thaliana*, 19 from *Oryza sativa* (rice), and 28 from *P. trichocarpa* (Supplementary Tables S7, S8). Only the highly conserved SBP domains (76 aa) sequences were used for phylogenetic analysis (Riese et al., 2007; Li et al., 2013; Zhang et al., 2016). As a result, these SPL proteins were clustered into 9 groups (I-IX), each of which included at least one BlSPL (Figure 2). Remarkably, all the *BlSPL* genes, except *BlSPL14*, that clustered into group IV to IX were putative target genes of BlmiR156 (Table 1 and Figure 2). As reported in previous studies, this phylogenetic tree indicated the evolutionary diversification and differentiation of *BlSPL* genes as well as other plant *SPL* genes.

**Figure 3:**
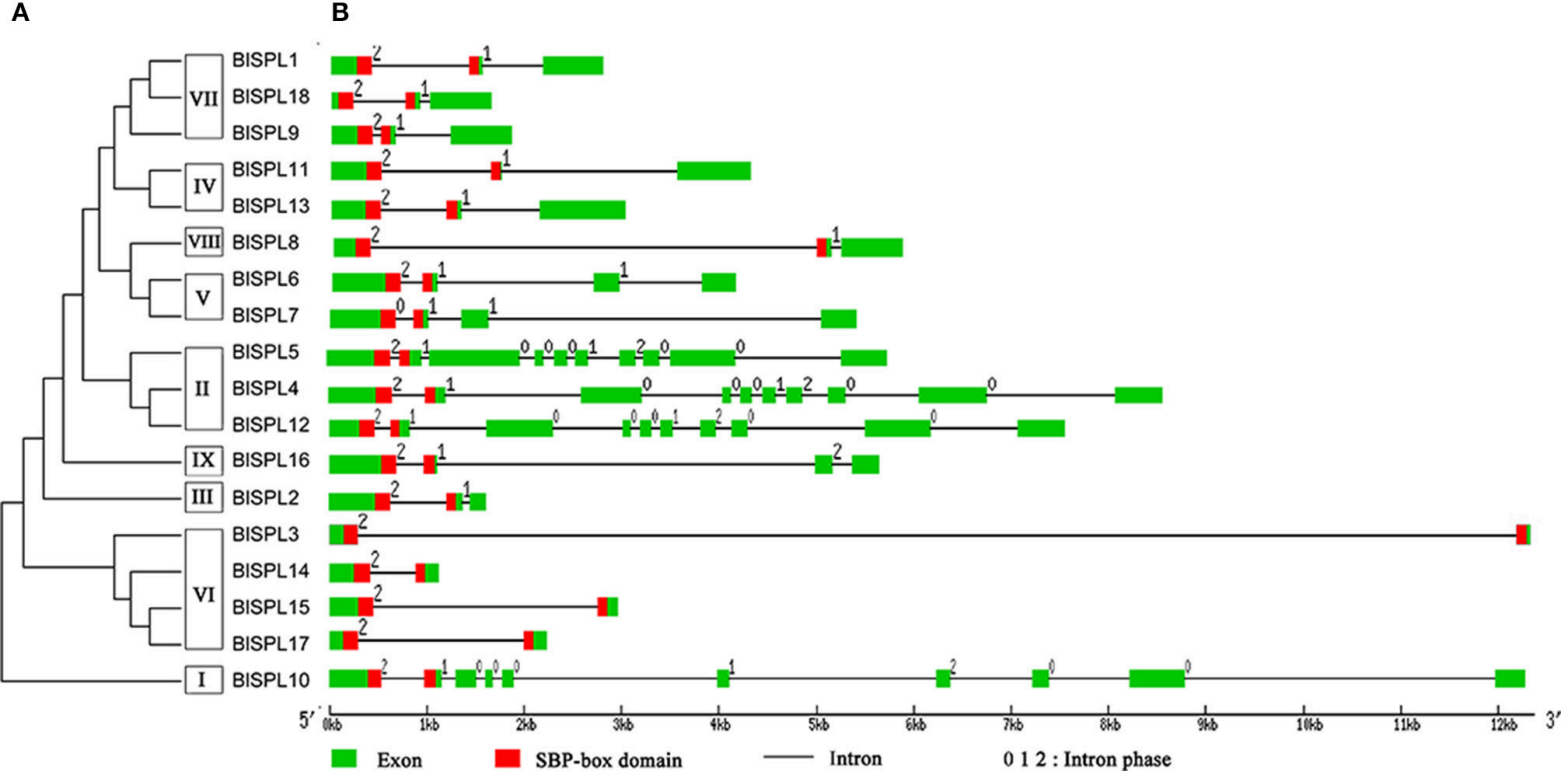
Phylogenetic analysis and intron/exon structures of *BlSPL* genes. **(A)** A phylogenetic tree was constructed with *B. luminifera* SBP domain protein sequences. **(B)** Exons and introns are indicated by green boxes and black horizontal lines, respectively.

By aligning the coding sequences to corresponding genomic sequences, intron/exon structures of all 18 *BlSPL* genes were also analyzed (Figure 3). As shown in Figure 3, high variation in the number of introns was revealed, which ranges from one (*BlSPL3, BlSPL14, BlSPL15*, and *BlSPL17*) to 9 (*BlSPL4, BlSPL5, BlSPL10*, and *BlSPL12*). Based on the BlSPL tree (Figure 3A), group I and II proteins contain 9 introns, group III and IV contain 2, group V contains 3, group VI contains 1, group VII, and VIII contain 2, group IX contains 3 introns (Figure 3B). In general, the *BlSPL* genes shared a similar intron/exon structure within the same group. For example, *BlSPL* genes within group VI had only one intron, whereas those within group II contained nine introns. In addition, all the SBP domains of *BlSPL* genes were separated by the first intron, which were highly variable in length among the *BlSPL* genes.

### Identification of conserved motifs in BLSPLs

**Figure 4:**
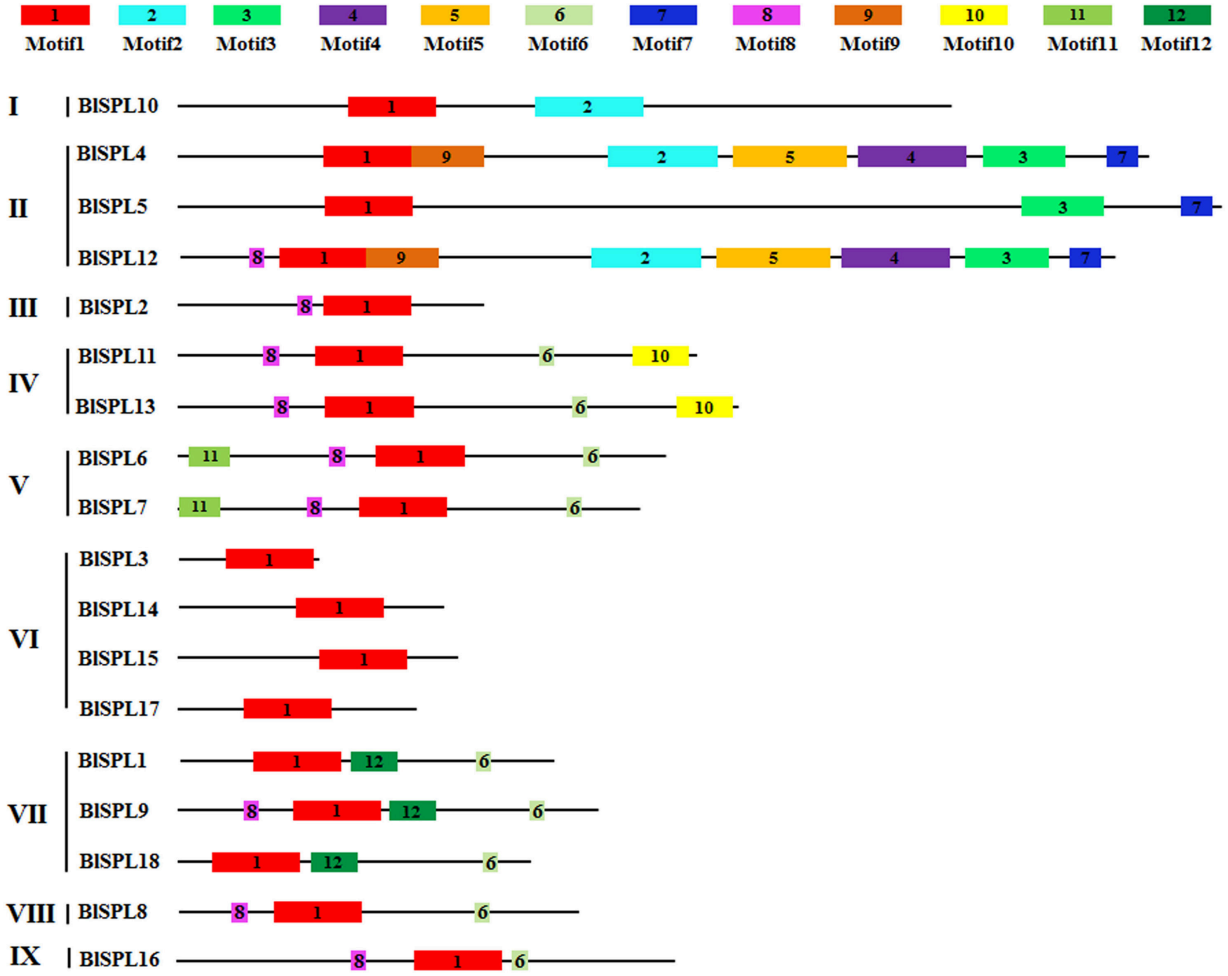
Distribution of conserved motifs in BlSPLs. Motifs represented with boxes are identified using MEME online tool. The number in boxes (1–12) represents motif 1-motif 12, respectively. The position and length of each colored box represents the actual motif size.

By using the MEME web server, a total of 12 conserved motifs for 18 BlSPLs were identified (Figure 4, Table 2). The number of motifs in each BlSPL varies from 1 to 8, and most BlSPLs shared similar motif profiles within the same group (Figure 4). Among these motifs, motif 1 is actually the SBP domain, which exists in all BlSPLs analyzed. Motif 6 existed in Group VI-V and VII-IX BlSPLs contains the complementary sequence of miR156, suggesting the posttranscriptional regulation of Group VI-V, VII-IX BlSPLs by miR156. In addition, motif 8 also widely exists in several SPL groups. Interestingly, several motifs are found to be group-unique, such as motif 12 specifically existing in group VII, and motifs 3 (ANK domain) and 7 are found exclusively in group II (Figure 4). Functions of these group-unique motifs are currently unclear, but should be tightly related to specific roles of SPLs in the group.

**Table 2:** Consensus sequences of 12 motifs identified in BlSPLs.

**Motif**	**Length (aa)**	**Consensus sequence**
1	80	PM**C**QVDN**C**KEDLSNAKDY**H**RRHK**VC**EV**H**S**K**SGKALVANQMQ**RFCQQC**SR**FH**PLSE**FD**EG**K**R**SCR**RR**L**AG**HN**R**RRR**KTQPE
2	109	AQSR**T**D**RI**V**FKL**FGKE**P**ND**FP**LV**LR**AQ**I**LD**WL**SHS**P**TDI**E**S**YIRPGC**II**L**TIYLRQAESA**W**EE**L**CYDLSSSLNRLLDVSDDSFWRT**G**WVYIRVQHQIAFIYN**G**QVVIDT
3	74	FL**F**R**P**DVK**GP**A**G**L**TPLH**I**AA**GKD**GS**ENVL**DALT**D**DP**KMV**G**IEA**W**KSAR**D**ST**G**STPED**YA**RLRGHF**SY**IH**LV**QK**K**
4	105	**EAK**T**QA**M**DFVHE**M**G**W**LLHRS**NA**K**F**RL**S**HLDPN**Q**DLFP**F**KRFKWLMEFSMDHDWCAVV**KKLLN**IL**FECV**V**DA**GDHPS**VV**LAL**LDLS**LLHRAVRRNCR**PM**VELLLR**F
5	112	**KPIA**V**S**V**SER**V**QF**V**VK**VF**NL**S**R**SSA**RLLCA**Q**EG**K**YL**VQ**E**TCY**D**LMDGA**D**TATE**H**G**ELQC**LS**F**P**CSIP**N**V**T**GRGFIE**VE**DH**CL**SSSFFPFIVAE**QE**VCSEI**CM**LE**G**A**I**E**VA**ET**
6	14	DSTC**AL**S**LLS**NQTW
7	31	YQ**P**AML**S**MVAI**A**A**VCVCV**A**L**LFKS**SP**EVLF**V**
8	15	IG**L**K**LG**KRTYFEDLC
9	61	**T**V**VNG**G**ALND**EKG**S**S**YLLISLLRILSNMHSN**NS**DQT**K**DQDLLSHLLR**N**LA**GL**A**GTVD**G**R**NI**
10	56	I**TD**RM**FQ**G**SD**C**LN**S**N**NRN**S**C**E**N**GPT**M**D**L**LQLSS**Q**LQRVE**H**QR**QFM**Q**M**KQ**DNDAF**C**C
11	19	**M**E**WN**E**K**S**AS**Q**W**E**WE**N**L**FM**F**
12	29	**FLS**G**Y**Q**G**TTILT**FSS**PQILPQGAVV**S**SAW

### Posttranscriptional regulation of *BLSPL*s mediated by miR156

**Figure 5:**
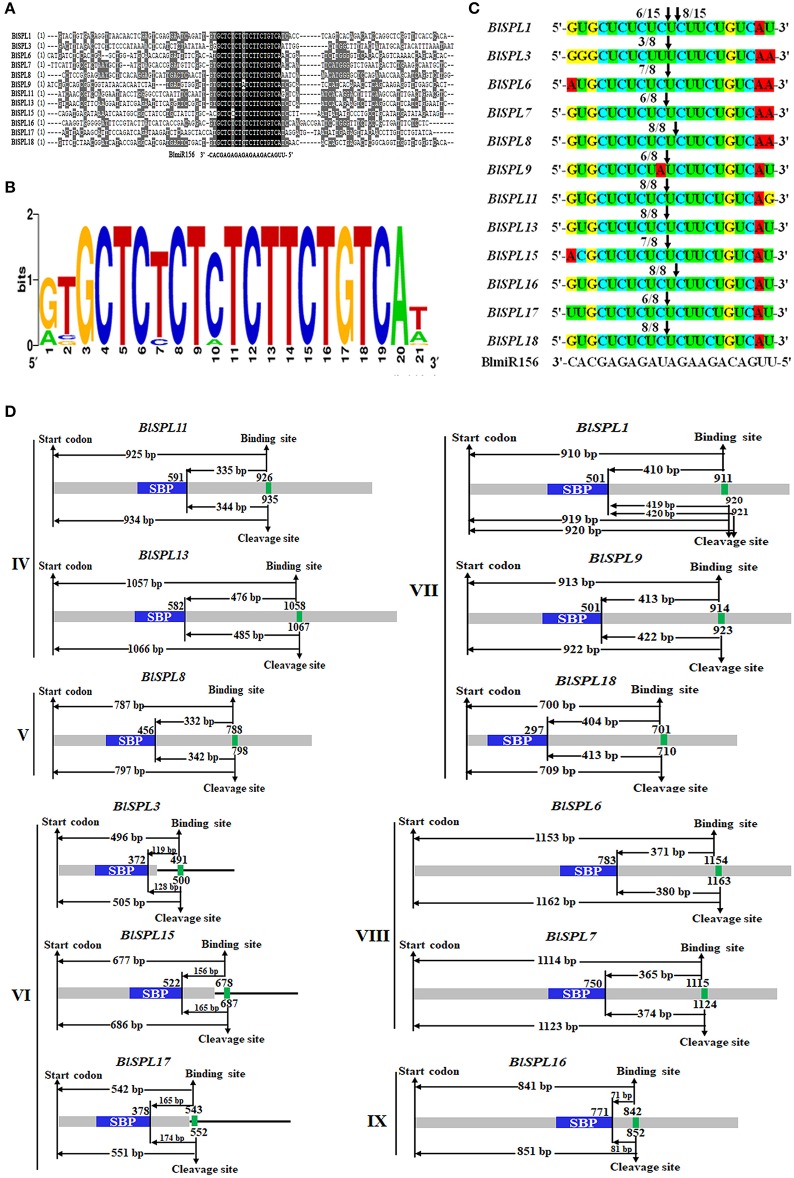
Analysis of miR156-mediated posttranscriptional regulation of *BlSPL*s. **(A)** Sequence alignment of miR156 complementary sequences of the *BlSPL*s. The 50 nucleotides upstream and downstream of the miR156 complementary sequences were included in the alignment. **(B)** Sequence logo of the miR156 complementary sequences of *BlSPL* genes. **(C)** Confirmation of the cleavage sites in twelve *BlSPL* transcripts by RLM 5'-RACE. Vertical arrows indicate the cleaved nucleotide position, with the frequency of clones shown. **(D)** Distances between important sites in sequences of twelve *BlSPL* genes. Important sites include start codon, SBP domain, and binding sites, cleavage sites of the miR156 complementary sequences with their nucleotide positions. Heavy gray lines represent open reading frames (ORFs). The lines flanking ORFs represent 3'-UTR. SBP domains are indicated in blue. MiR156 complementary sequences of *BlSPL* genes are indicated in green. Distances between these sites are measured and indicated by base pair (bp).

It has been confirmed that a subset of *SPL* genes are regulated by miR156 at posttranscriptional level in plant (Schwarz et al., 2008; Wang et al., 2009, 2011; Jiao et al., 2010; Wang and Wang, 2015; Tripathi et al., 2017). With the psRNATarget online tool, a total of 12 *BlSPL*s were predicted to be targeted by miR156 (Figure 5).The complementary sites of miR156 are in coding regions for nine *BlSPL*s (*BlSPL1, BlSPL6, BlSPL7, BlSPL8, BlSPL9, BlSPL11, BlSPL13, BlSPL16*, and *BlSPL18*) belonging to group IV, V, VII, VIII, and IX, whereas it locates in the 3'-UTR of *BlSPL3, BlSPL15* and *BlSPL17* within group VI (Figure 5D). Sequence alignment indicated that the miR156 complementary sites were quite conserved across these *BlSPL* genes (Figure 5A), and the sequence divergence was mainly restricted to the first, second and last nucleotide of complementary sequences (Figure 5B). It means that the complementary sites are actually under strong selection pressure during the evolution, even for those locating in the 3'-UTR. These results are consistent with the previous studies from other plants, suggesting the importance of miR156-mediated posttranscriptional regulation for functions of *SPL* genes in plant.

In order to validate the cleavage of the *BlSPLs* transcript by miR156, RLM 5'-rapid amplification of the cDNA ends (5'-RACE) was performed for all twelve *BlSPL*s. The result indicated that two cleavage sites of miR156 were detected in the complementary sequences of these twelve *BlSPL*s (Figure 5C). One of the cleavage sites was present between the 10th and 11th nucleotides of the complementary sequences in ten *BlSPL*s (*BlSPL1, BlSPL3, BlSPL6, BlSPL7, BlSPL9, BlSPL11, BlSPL13, BlSPL15, BlSPL17*, and *BlSPL18*), while the other cleavage site was located between 11th and 12th nucleotides of the complementary sequences in three *BlSPL*s (*BlSPL1, BlSPL8* and *BlSPL16*) (Figure 5C). Interestingly, both of these two cleavage sites were detected in *BlSPL1*. Thus, all these results validate the posttranscriptional regulation of the *BlSPL* genes by miR156 in *B. luminifera*, and imply that these *BlSPL*s might be regulated by different miR156 genes through different cleavage sites.

Moreover, by comparing the distances between important sequence sites of these *BlSPL* genes, three types of distance between SBP domain and complementary site were revealed. It could be roughly classified into short-distance (less than 200 bp), medium-distance (approximately 350 bp) and long-distance (more than 400 bp) (Figure 5D). Similar distance type was observed for the *BlSPL* genes within the same phylogenetic groups. For instance, the *BlSPL3, BlSPL15*, and *BlSPL17* in group VI have the short-distance type, whereas the *BlSPL*s of group VII have the long-distance type (Figure 5D). This implies that a common ancestral gene may have experienced different evolutionary events resulting in the differentiation of these miR156-regulated *BlSPL* genes.

### Cis-elements in the promoter regions of *BLSPL* genes

**Figure 6:**
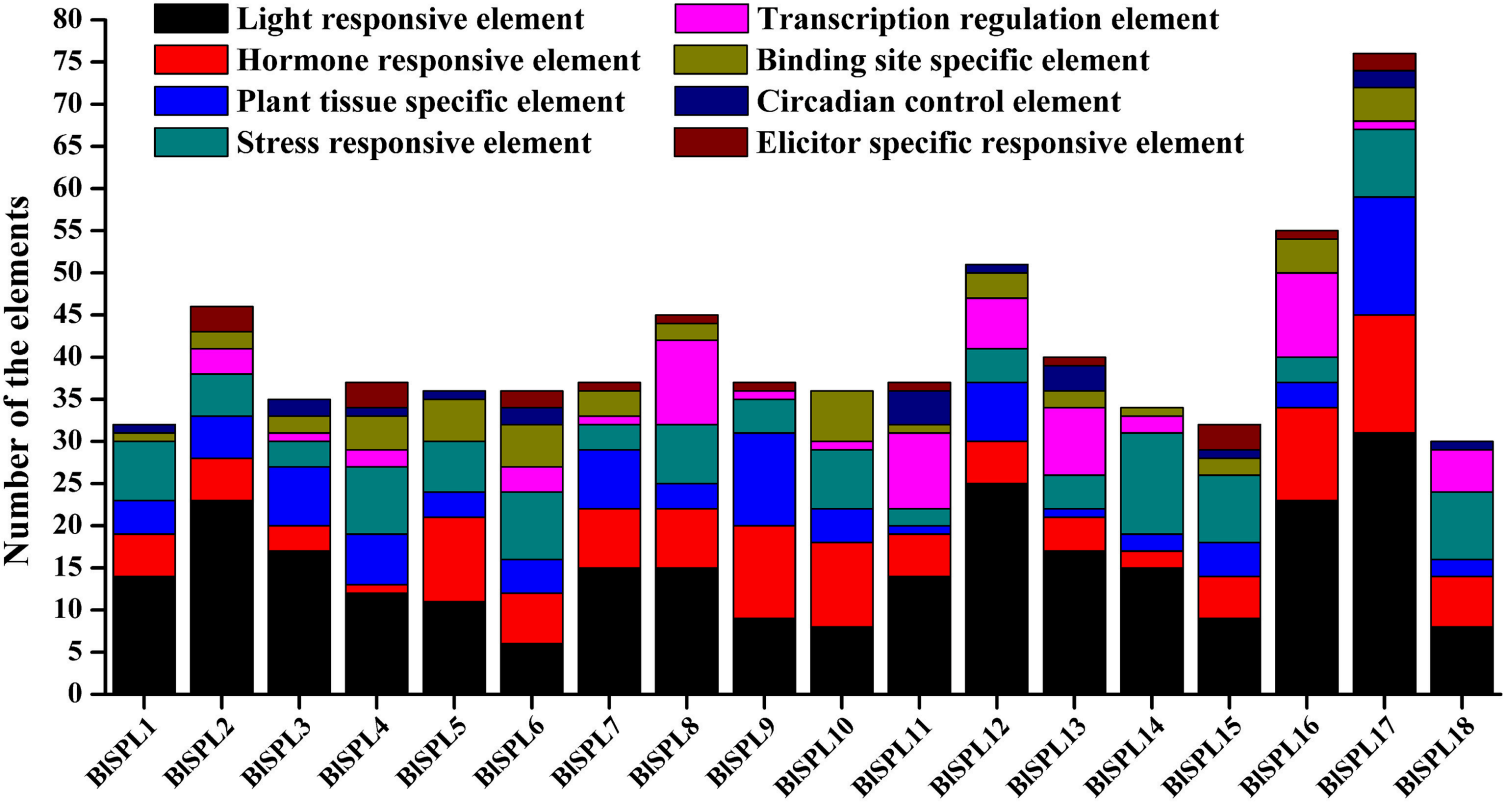
*Cis*-elements in promoter regions of 18 *BlSPL*s. Elements were identified in the 1500 bp sequences upstream of the start codon of *BlSPL* genes using PlantCARE database. The elements associated with specific functions are indicated by different colors for each gene.

Cis-elements play important roles in the regulation of gene transcription during plant growth, development, and stress responses. To understand the transcriptional regulation mechanisms, the cis-elements in promoter regions of *BlSPL* genes were identified through PlantCARE database. Except for the common cis-acting elements such as CAAT-box and TATA-box, many cis-elements were identified in promoter regions of 18 *BlSPL* genes (Supplementary Table S9). According to their putative functions, these elements were categorized into eight classes, which are shown in the Figure 6. As the results, light-responsive elements had the largest number and were present in all *BlSPL* gene promoter regions (Supplementary Table S9, Figure 6). And, the hormone responsive elements, plant tissue-specific elements, and stress responsive elements are also found to be present in promoter regions of all *BlSPL* genes (Supplementary Table S9, Figure 6). In addition, other rarely distributed cis-elements in *BlSPL*s were found to be functionally involved in transcription regulation, circadian control, protein binding, elicitor responsiveness (Supplementary Table S9, Figure 6). Thus, the transcription of *BlSPL* genes could be regulated by various environmental and developmental changes, which implied that *BlSPL* genes were involved in important physiological processes and developmental events. Furthermore, there no very similar cis-elements distribution was observed among these *BlSPL* genes, even for those *BlSPL*s in the same phylogenetic group (Supplementary Table S9, Figure 6). These observations suggest that the differentiations in the promoter regions may promote the neofunctionalization of *BlSPL* genes during their divergence and evolvement.

### Expression profiles of the *BLSPL* genes and Blmir156 in different tissues/organs of *B. luminifera*

**Figure 7:**
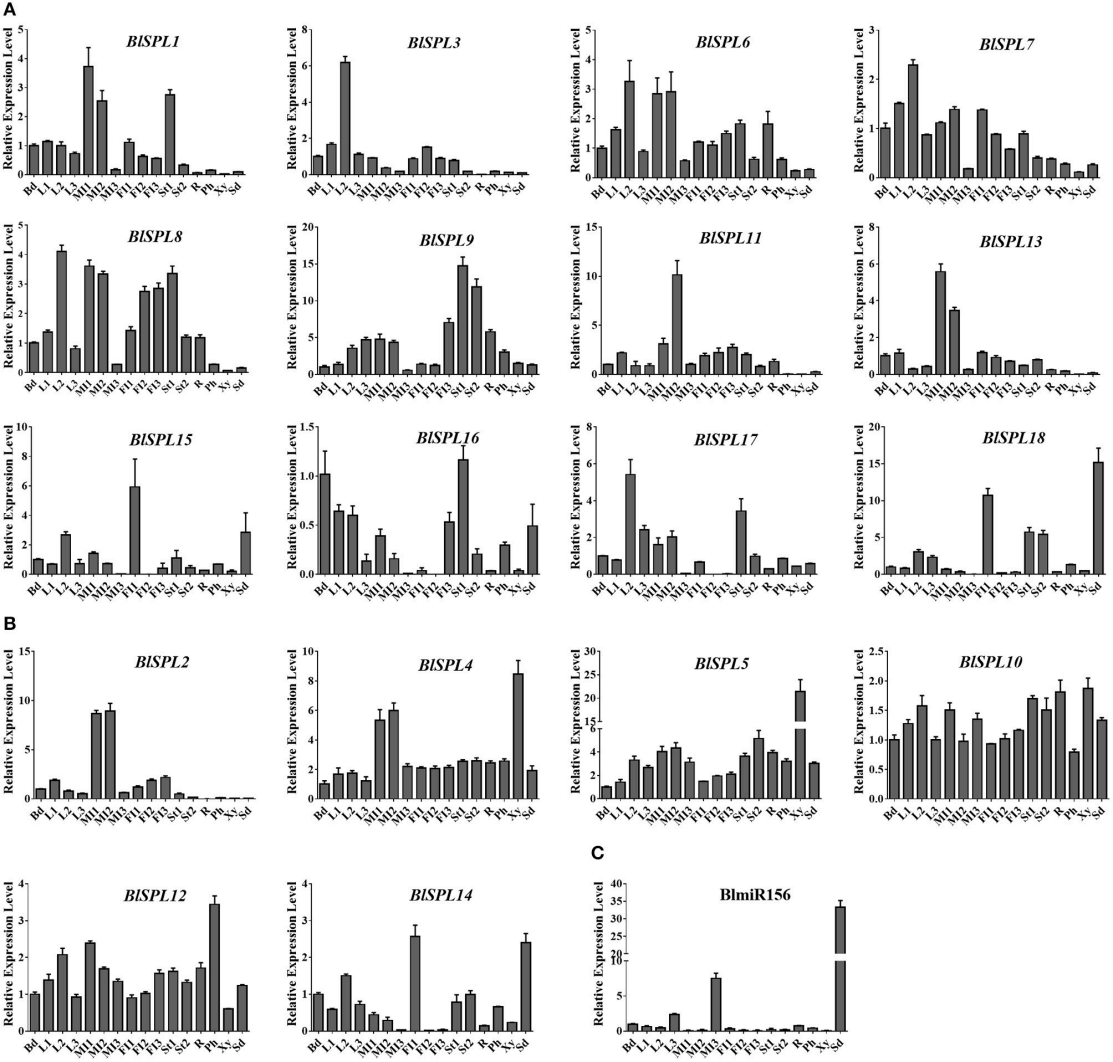
Expression profiles of *BlSPL*s and mature BlmiR156 in different tissues of *B. luminifera*. **(A)** Expression patterns of BlmiR156-targeted *BlSPL*s. **(B)** Expression patterns of BlmiR156-nontargeted *BlSPL*s. **(C)** Expression patterns of BlmiR156. Fold changes of transcript levels in buds (Bd), unexpanded leaves (L1), newly expanded leaves (L2), mature leaves (L3), male inflorescence at early stage (MI1), male inflorescence at mid stage (MI2), male inflorescence at late stage (MI3), female inflorescence at early stage (FI1), female inflorescence at mid stage (FI2), female inflorescence at late stage (FI3), young stems (St1), mature stems (St2), root (R), phloem (Ph), xylem (Xy), Seed (Sd) of *Betula* plants are shown. Transcript levels in buds were arbitrarily set to 1 and the levels in other tissues were given relative to this. Error bars represent standard deviations of mean value from three biological replicates and four technical replicates.

In order to explore the possible roles of *BlSPL*s in development of *B. luminifera*, the expression profiles of these 18 *BlSPL* genes in various tissues/organs were investigated using the quantitative real-time RT-PCR method (qRT-PCR). In general, the expression patterns of *BlSPL* genes can be classified into two types according to their expression profiles (Figure 7). The minority of *BlSPL* genes, including *BlSPL4, BlSPL5, BlSPL10*, and *BlSPL12*, were constitutively expressed in all tissues/organs examined (Figure 7B). And, all these genes are *BlSPL* genes without miR156 complementary sites (termed as BlmiR156-nontargeted *BlSPL* genes). The remaining majority of *BlSPL* genes, most of which are *BlSPL* genes with miR156 complementary sites (termed as BlmiR156-targeted *BlSPL* genes), exhibited a more differentiated expression pattern in different tissues or organs (Figure 7A). This difference implies distinct roles of BlmiR156-targeted and nontargeted *BlSPL* genes in development of *B. luminifera*. In addition, similar expression patterns were observed among those genes belonging to same group, such as *BlSPL6*/*7* in group V, *BlSPL4*/*5*/*12* in group II, *BlSPL14*/*15* in group VI (Figure 7), suggesting the redundant functions of these *BlSPL* genes. On the other hand, it was particularly worthy to note that many *BlSPL*s were expressed at a relatively low level in seeds compared with their expressions in other tissues or organs analyzed (Figures 7A,B). Furthermore, the transcript levels of mature BlmiR156 in different tissues or organs of *B. luminifera* were also investigated by qRT-PCR. According to its expression pattern, the mature BlmiR156 was mainly accumulated in seeds, which was approximately four times of the second highest level in male inflorescences at late stage (MI3) (Figure 7C). In contrast, in many tissues or organs, such as the buds, young leaves (L1 and L2), female inflorescences, stems, phloem and xylem, the transcript levels of mature BlmiR156 were rather low (Figure 7C).

### Expression profiles of the *BLSPL* genes and BlmiR156 with age in *B. luminifera*

**Figure 8:**
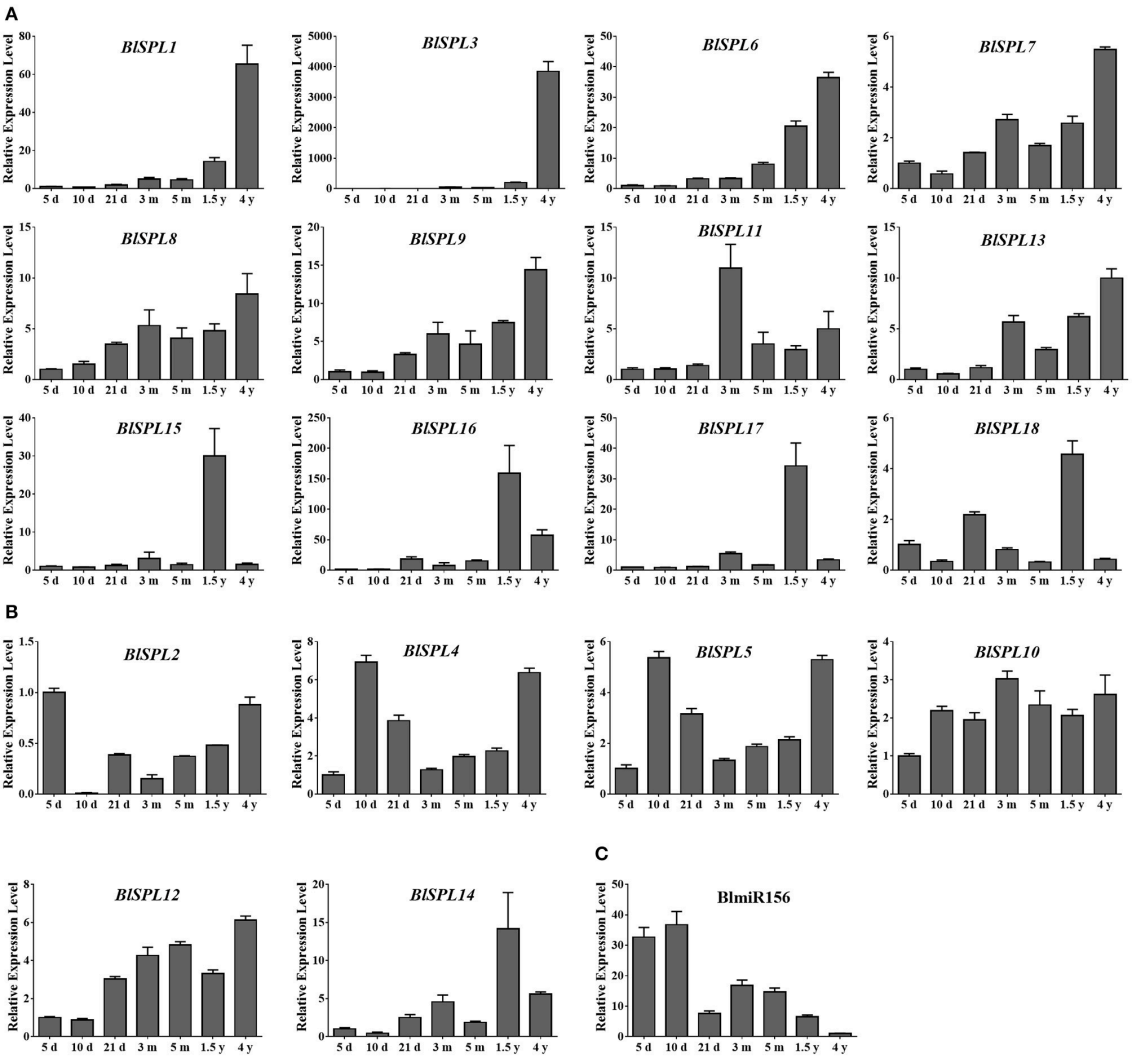
Expression profiles of *BlSPL*s and mature BlmiR156 with age in *B. luminifera*. **(A)** Expression patterns of BlmiR156-targeted *BlSPL*s. **(B)** Expression patterns of BlmiR156-nontargeted *BlSPL*s. **(C)** Expression patterns of BlmiR156. Fold changes of transcript levels in 5- (5 d), 10- (10 d) and 21-day-old (21 d) seedlings, and 3- (3 m), 5- (5 m), 1.5-year (1.5 y), and 4-year-old (4 y) plants are shown. Transcript levels of *BlSPL*s in 5-day-old (5 d) seedlings were arbitrarily set to 1 and the levels in other tissues were given relative to this. Transcript levels of BlmiR156 in 4-year-old (4 y) plants were arbitrarily set to 1 and the levels in other samples were given relative to this. Error bars represent standard deviations of mean value from three biological replicates and four technical replicates.

MiR156 regulated *SPL* genes play vital roles in regulation of plant vegetative phase change (Wang et al., 2009, 2011). To investigate the roles of *BlSPL* genes in vegetative phase change of *B. luminifera*, the expression patterns of *BlSPL* genes and mature BlmiR156 were examined in seedlings or fully expanded leaves of plants with different ages. Similar as in different tissues or organs, two distinct expression patterns of these *BlSPL* genes were observed (Figure 8). Most of the BlmiR156-targeted *BlSPL* genes, especially for *BlSPL1, BlSPL3*, and *BlSPL6*, were dramatically up-regulated with age in plants (Figure 8A), while most of the BlmiR156-nontargeted *BlSPL* genes showed a constitutive expression pattern (Figure 8B). In contrast, the mature BlmiR156 was strongly accumulated in 5- and 10-day-old seedlings, and dramatically down-regulated in 1.5-year-old and 4-year-old plants (Figure 8C). These results indicate the functional differentiation between BlmiR156-targeted and nontargeted *BlSPL*s, and also suggest important roles of these BlmiR156-targeted *BlSPL* genes in vegetative phase change of *B. luminifera*.

### Protein interaction between BLSPLs and DELLA proteins

**Figure 9:**
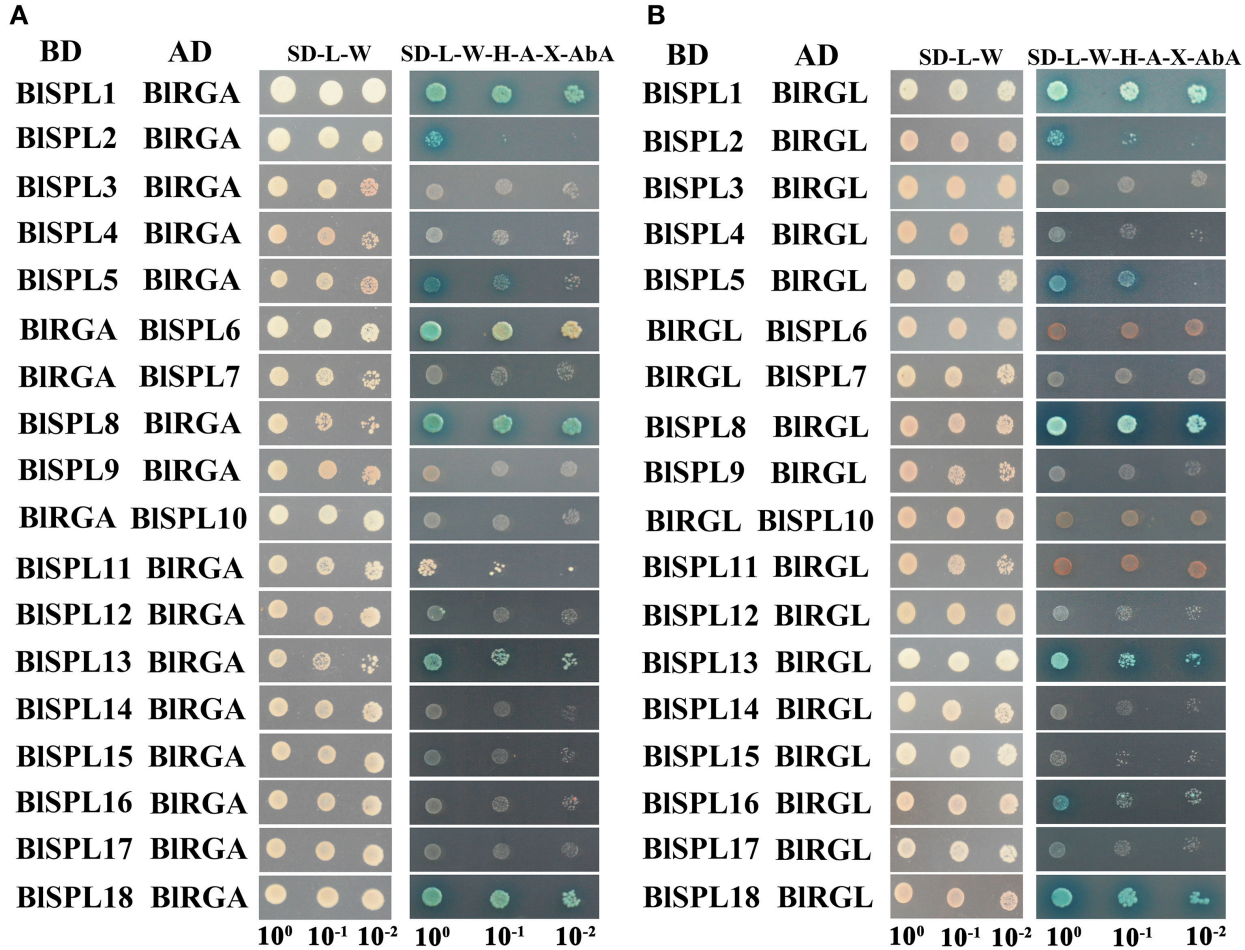
Interactions of BlSPLs with DELLA proteins. **(A)** Interaction between BlSPLs and BlRGA revealed by yeast two-hybrid. **(B)** Interaction between BlSPLs and BlRGL revealed by yeast two-hybrid. BD, GAL4 binding domain; AD, GAL4 activation domain. Interactions were examined on the SD-Leu-Trp-His-Ade plates supplemented with 40 μg/ml X-α-gal and 125 ng/ml Aureobasidin A (SD-L-W-H-A-X-AbA). Yeast grown on the SD medium without leucine and tryptophan (SD-L-W) were used as a control. The yeast clones were grown with dilutions to 10^−1^ and 10^−2^.

The interactions between miR156-targeted SPL transcription factors and DELLA proteins have been suggested to be an important regulatory module for GA regulated floral transition in plant (Yu et al., 2012; Yamaguchi et al., 2014). In our study, yeast two-hybrid assay was used to survey the interaction between BlSPL proteins and two DELLA proteins (BlRGA and BlRGL) of *B. luminifera*. As shown in Figure 9, similar interaction patterns were observed between BlSPLs and these two DELLA proteins. There six BlSPL proteins (BlSPL1/2/5/8/13/18) interact with both DELLA proteins, while the BlSPL6 only interact with BlRGA (Figure 9A), and BlSPL16 only interact with BlRGL (Figure 9B). It is worth to note that not only BlmiR156-targeted *BlSPL* genes but also nontargeted *BlSPL* genes (BlSPL2 and BlSPL5) could interact with DELLA proteins. On the other hand, the interaction strength between these BlSPL proteins and DELLA proteins are obvious different. For example, BlSPL1, BlSPL8, and BlSPL18 tightly interact with BlRGA and BlRGL. Conversely, the interaction between BlSPL2 and BlRGA as well as BlSPL16 and BlRGL are very weak (Figure 9). The strength differences between these interactions were also validated through β-galactosidase activity assay (Supplementary Figure S3). These results indicate interaction with DELLA proteins is possibly significant for functions of these *BlSPL* genes. And, the BlmiR156-targeted *BlSPL* genes may also participate in GA regulated flowering in *B. luminifera*.

Additionally, the expressions of these two *DELLA* genes were also investigated in different tissues/organs and plants with different ages. As shown in Supplementary Figure S4, different expression patterns were revealed for these two *DELLA* genes. The *BlRGA* was highly expressed in xylem (Xy) and male inflorescence at early stage (MI1) (Supplementary Figure S4A), and reached to highest expression level in 21-day- old plants (Supplementary Figure S4C). Whereas, the *BlRGL* was evenly expressed in most tissues/organs examined (Supplementary Figure S4B), and gradually up-regulated with age in plants (Supplementary Figure S4D). For those interacted *BlSPL* genes, further correlation analysis indicated that the expression of *BlSPL5* was significantly correlated with the expressions of *BlRGA* and *BlRGL* in different tissues/organs, while the expressions of *BlSPL1, BlSPL8*, and *BlSPL13* were merely significantly correlated with that of *BlRGL* in plants with different ages (Supplementary Table S10). It means that these *BlSPL* genes might be co-expressed with the *DELLA* genes in specific tissue/organ or developmental stage, which may determine their protein interactions and functions.

## Discussion

SPLs are one family of the plant-specific transcription factors featured with a highly conserved SBP domain, which is capable of binding to the promoters of floral meristem identity gene *SQUAMOSA* and its homologs (Klein et al., 1996). The *SPL* genes have been identified from many plant species, such as *Arabidopsis* (Cardon et al., 1999), rice (Yang et al., 2008), soybean (Tripathi et al., 2017), *Populus* (Li and Lu, 2014), chrysanthemum (Song et al., 2016), and pepper (Zhang et al., 2016). However, no genome-wide identification of *SPL* gene family has been reported in *B. luminifera*. In this study, genome-wide identification of the *SPL* gene family was performed in *B. luminifera* for the first time. As a result, 18 *SPL* genes were identified in the genome of *B. luminifera*, which is similar to the number of *SPL* genes in *Arabidopsis* (16) and rice (19), but much less than that of *P. trichocarpa* (28), or that of moso bamboo (32) (Pan et al., 2017). It implies that the *SPL*s of different plants evolved in a species-specific manner, which is possibly influenced by different gene duplication events.

Based on the phylogenetic analysis, the *BlSPL* gene family was divided into nine groups (I-IX) (Figure 2). And, the *BlSPL* genes grouped tightly with *AtSPL* and *PtSPL* genes, reflecting the fact that these *SPL* genes may diverge more recently from a common ancestor. Gene structure analyses showed that *BlSPL* genes shared similar intron/exon structures within the same phylogenetic group (Figure 3), as described previously in cotton (Cai et al., 2018), apple (Li et al., 2013), tomato (Salinas et al., 2011), and rice (Xie et al., 2006). Moreover, most BlSPLs of the same phylogenetic group also shared similar motifs (Figure 4), which showed that these genes within the same phylogenetic group may have similar functions in *B. luminifera*. Interestingly, except for the conserved motifs present in most BlSPLs, several unique motifs were also found in specific phylogenetic groups (Figure 4), such as motif 4 in group II, motif 10 in group V and motif 12 in group VII. These unique motifs are probably important for their specified functions, indicating that the many *BlSPL* genes may have undergone functional differentiation and neofunctionalization with the divergence of different lineages.

MiR156-mediated posttranscriptional regulation has been considered as an important mechanism for the functions of a subset of *SPL*s (Gandikota et al., 2007; Wang et al., 2009, 2017; Gou et al., 2011, 2017; Kim et al., 2012). In *Arabidopsis*, 10 of 17 *AtSPL*s are targets of miR156 (Rhoades et al., 2002). Also, 11 of 19 *SPLs* in rice (Xie et al., 2006), 18 of 28 *SPLs* in *Populus* (Li and Lu, 2014), 10 of 15 *SPLs* in *Citrus* (Shalom et al., 2015) and 12 of 18 SPLs in *Ziziphus jujube* (Shao et al., 2017), were reported to be targeted by miR156. In our study, target prediction and experimental validation showed that 12 *BlSPL*s in groups IV-IX were regulated by miR156 (Figure 5). In addition, two kinds of the complementary sites of miR156 were detected in these *BlSPL*s, one of which locates in 3' UTR of *BlSPL*s in group VI, whereas the other one locates in the coding region. High conservation of the miR156 complementary sequences across all miR156-targeted *BlSPL*s was also observed in both coding regions and 3' UTR (Figures 5A,B). It is consistent with the results from *SPL*s of *Arabidopsis, Salvia miltiorrhiza, Populus, Ziziphus jujuba* and *Citrus*, which suggests that the miR156-mediated posttranscriptional regulation of *SPL* genes should be highly conserved in plants.

Cis-elements in the promoter regions are closely correlated with gene expression and/or tissue specificity in development and responses to stress. Many cis-elements were found in the promoter regions of *BlSPL*s (Figure 6, Supplementary Table S9), and lots of these cis-elements are associated with light-responsiveness, hormone responsiveness, transcription regulation and stresses responses. Similar cis-elements were also identified in moso bamboo and upland cotton, which suggests that the expressions of *SPL* genes may be regulated by light, stresses and/or phytohormones (Pan et al., 2017; Cai et al., 2018). Interestingly, no similar cis-elements distribution was shown in promoter regions of *BlSPL*s within the same phylogenetic group (Figure 6), implying the function divergences of *SPL* genes was not only reflected in the coding regions, but also occurred in the promoter regions.

In order to further explore the potential roles of *BlSPL* genes in growth and development of *B. luminifera*, the expression profiles of *BlSPL* genes were analyzed in 16 different tissues/organs. Considering the correlation between *BlSPL*s and miR156, the expression profile of mature BlmiR156 was also analyzed accordingly. Different expression patterns of the *BlSPL* genes were revealed (Figure 7). A few *BlSPL* genes (*BlSPL4, BlSPL5, BlSPL10*, and *BlSPL12*) presented a constitutive expression pattern in all tissues or organs examined, the remaining majority exhibited a development- and tissue-dependent expression patterns. On the other hand, it is worthy to note that the differentiated expression pattern was usually observed in the BlmiR156-targeted *BlSPL* genes, while more than half of the BlmiR156-nontargeted *BlSPL* genes tended to exhibit the constitutive expression pattern. Similar expression differences between miR156-targeted and nontargeted *SPL* genes have been revealed in apple, soybean, *Brassica napus* and *S.miltiorrhiza* (Li et al., 2013; Zhang et al., 2013; Cheng et al., 2016; Tripathi et al., 2017). This means that miR156-targeted and nontargeted *SPL* genes experienced divergent evolution with differentiated expression patterns, suggesting their distinct roles in development and growth of plants. Moreover, the BlmiR156 showed a differentiated expression pattern in different tissues/organs, with highest expression level in seeds and comparative low level in most of the remaining tissues/organs (Figure 7). Notably, most of the BlmiR156-targeted *SPL* genes exhibited rather low expressions in seeds. This is consistent with the results from apple, in which miR156 was also highly expressed in seeds of young and mature fruit, and many miR156-targeted *MdSPL* genes showed extremely low expression levels in seeds (Li et al., 2013). If taken the seeds as the plants with minimum physiological age, it implies the tight correlation of miR156/*SPL* module with vegetative phase changes in trees.

Because of the important regulatory roles of miR156/*SPL* module in vegetative phase change, the expressions of *BlSPL*s and BlmiR156 were evaluated in seedlings/plants with different physiological ages. As expected, most BlmiR156-targeted *BlSPL* genes were dramatically up-regulated with ages, whereas most BlmiR156-nontargeted *BlSPL* genes tend to be constitutively expressed at different ages (Figure 8). Meanwhile, the expression of BlmiR156 was obviously decreased with ages (Figure 8), which is correlated negatively with the expression patterns of those BlmiR156-targeted *BlSPL* genes. Such age-dependent expression patterns of miR156 and miR156-targeted *SPL* genes have also been reported in *Arabidopsis, Arabis alpine* and *Populus* (Wang et al., 2011; Bergonzi et al., 2013; Xu et al., 2016). Further functional studies have indicated the important roles of miR156/SPL module in phase transition. In *Arabidopsis*, constitutive expression of miR156 causes prolonged expression of juvenile vegetative traits and delayed flowering (Wu and Poethig, 2006). *AtSPL3*/*4*/*5* directly activate *LEAFY* (*LFY*), *FRUITFULL* (*FUL*), and *APETALA1* (*AP1*) to redundantly promote flowering in *Arabidopsis* (Yamaguchi et al., 2009). In addition, *AtSPL9* and *AtSPL10* also act redundantly to promote the transcription of miR172, which further promotes the transition from the juvenile to the adult phase (Wu et al., 2009). Similar functions of miR156 and *SPL* genes have also been revealed in Chinese cabbage (Wang et al., 2014), rice (Xie et al., 2006) and perennial plants such as the tree *P x Canadensis* (Wang et al., 2011), *Cardamine flexuosa* (Zhou et al., 2013). These evidences show that the miR156/SPL module plays a conserved role in regulating vegetative phase change in diverse plant species. Thus, these miR156-targeted *BlSPL*s must also have important functions during vegetative phase change of *B. luminifera*, and could be considered as molecular markers for reaching to reproductive phase. Additionally, it was also noticed that four BlmiR156-targeted *BlSPL* genes (*BlSPL15*-*BlSPL18*) showed their highest expression levels at 1.5-year-old, when the *B. luminifera* plants start to flowering. While, the other BlmiR156-targeted *BlSPLs* (except for *BlSPL11*) reached to their highest expression level at 4-year-old (Figure 8). This expression difference suggested that these miR156-targeted *BlSPL* genes may not only have overlapping functions, but also play important roles in different stages of phase transition.

DELLA proteins, act as central transcriptional repressors of GA responses, have been found to interact and repress SPL proteins activities. The physical interaction between SPL and DELLA is thought to be an integrator of the age and GA pathways in flowering (Yu et al., 2012). The widespread interactions between DELLAs and miR156-targeted SPLs have been confirmed in *Arabidopsis* by yeast two-hybrid. For instance, RGA could interact with AtSPL2, AtSPL9 and AtSPL11, and RGL1 interacts with AtSPL2, AtSPL10, and AtSPL11 (Yu et al., 2012). Consistently, the interactions between BlRGA and miR156-targeted BlSPLs (BlSPL1/6/8/13/18), as well as BlRGL and miR156-targeted BlSPLs (BlSPL1/8/13/16/18), were also detected in this study (Figure 9). However, not all miR156-targeted BlSPLs could interact with these two DELLA proteins, and the interaction strengths were apparently varied in different combinations (Supplementary Figure S3). It means the interactions between DELLA proteins and SPLs are conserved regulation mechanisms for flowering induction in plants, but not all miR156-targeted *SPL* genes take part in this process. More interestingly, two miR156-nontargeted BlSPLs (BlSPL2 and BlSPL5) were also revealed to interact with these two DELLA proteins, which were reported for the first time in our study. This implies that interactions between DELLA proteins and miR156-nontargeted *SPL*s may exert important functions in other GA regulated processes.

## Conclusion

This study here represents the first genome-wide characterization of *SPL* genes in *B. luminifera*. Comprehensive analyses were performed to characterize 18 predicted *BlSPLs*, including their sequence features, phylogeny, intron/exon structure, conserved motif, miR156-mediated posttranscriptional regulation, cis-elements in promoter regions and expression patterns. Furthermore, the interactions between BlSPLs and DELLA proteins were also investigated to explore their roles in GA regulated biological processes. The results showed that 18 BlSPLs were clustered into 9 groups, and most *BlSPL*s of the same group exhibit high conservation in sequence features, intron/exon structures, motif composition, and posttranscriptional regulation. Moreover, two distinct expression patterns were revealed for miR156-targeted and nontargeted *BlSPL* genes in different tissues/organs, suggesting the differentiated roles of these *BlSPL*s in development and growth of *B. luminifera*. The expression analysis in plants with different ages showed that miR156/SPL module may also play important roles in regulating vegetative phase change in *B. luminifera*. In addition, protein interaction assay indicated that *BlSPL* genes participate in GA regulated biological processes through physical interacting with DELLA proteins. These results provide useful information that facilitates further elucidation of the potential biological roles of *BlSPL* genes in *B. luminifera*.

## Author contributions

E-PL, X-YL, and Z-KT: conceived and designed the experiments; E-PL, X-YL, and M-YN: performed the experiments; H-HH, Z-KT, and J-HZ: contributed reagents, materials, analysis tools; E-PL and X-YL: analyzed the data; E-PL and X-YL: wrote the paper.

### Conflict of interest statement

The authors declare that the research was conducted in the absence of any commercial or financial relationships that could be construed as a potential conflict of interest.

## Supplementary material

Supplementary Figure S1 Autoactivation and toxicity test for the BD fused constructs and AD fused constructs. **(A)** Autoactivation and toxicity of BD-BlSPLs, and AD-BlRGA and AD-BlRGL. **(B)** Autoactivation and toxicity of BD- BlRGA, BD-BlRGL, and AD-BlSPL6/7/10. BD, GAL4 binding domain; AD, GAL4 activation domain. Autoactivation was detected on the SD-Leu-Trp-His-Ade plates supplemented with 40 μg/ml X-α-gal and 125 ng/ml Aureobasidin A (SD-L-W-H-A-X-AbA). Toxicity was examined on the SD medium without leucine and tryptophan (SD-L-W). The yeast clones were grown with dilutions to 10^−1^ and 10^−2^.

Supplementary Figure S2 Alignment of ANK/ANK-2 domain in BlSPL4, BlSPL5 and BlSPL10. The ANK/ANK-2 domain is marked by solid lines.

Supplementary Figure S3 The interaction strengths were determined through β-galactosidase activity assay. (A) The interaction strengths between BlSPLs and BlRGA. (B) The interaction strengths between BlSPLs and BlRGL. Empty, BD and AD vector without target genes, as a negative control.

Supplementary Figure S4 Expression profiles of *BlRGA* and *BlRGL*. **(A)** Expression patterns of *BlRGA* in different tissues/organs. **(B)** Expression patterns of *BlRGL* in different tissues/organs. **(C)** Expression patterns of *BlRGA* in plants with different ages. **(D)** Expression patterns of *BlRGL* in plants with different ages. The same samples as figure 7 and 8 were used for the expression profiling of *BlRGA* and *BlRGL*. Transcript levels in buds and 5-day-old (5 d) seedlings were arbitrarily set to 1 and the levels in other tissues were given relative to this. Error bars represent standard deviations of mean value from three biological replicates and four technical replicates.

Supplementary Table S1 The mapping coordinates of TBLASTN by querying AtSPLs and PtSPLs protein sequences against draft genome of *B. luminifera*.

Supplementary Table S2 The predicted and corrected gene models of *BlSPL* genes.

Supplementary Table S3 Primer names and their sequences used for gene cloning.

Supplementary Table S4 Primer names and their sequences used for 5' RLM-RACE.

Supplementary Table S5 Primer names and their sequences used for qRT-PCR.

Supplementary Table S6 Primer names and their sequences used for ORF cloning.

Supplementary Table S7 General information of *SPL* genes used for phylogenetic analysis.

Supplementary Table S8 Data sources of *SPL* genes used for phylogenetic analysis.

Supplementary Table S9 Cis-elements in the promoter region of 18 *BlSPL* genes.

Supplementary Table S10 The pearson's correlation coefficients between expressions of two *DELLA* and *BlSPL* genes.
