# Supplementary figures and images for: Molecular Characterization of SQUAMOSA PROMOTER BINDING PROTEIN-LIKE (SPL) Gene Family in Betula luminifera
Source: Front Plant Sci. 2018 May 4;9:608. doi: 10.3389/fpls.2018.00608 (PMC5945835; doi:10.3389/fpls.2018.00608)

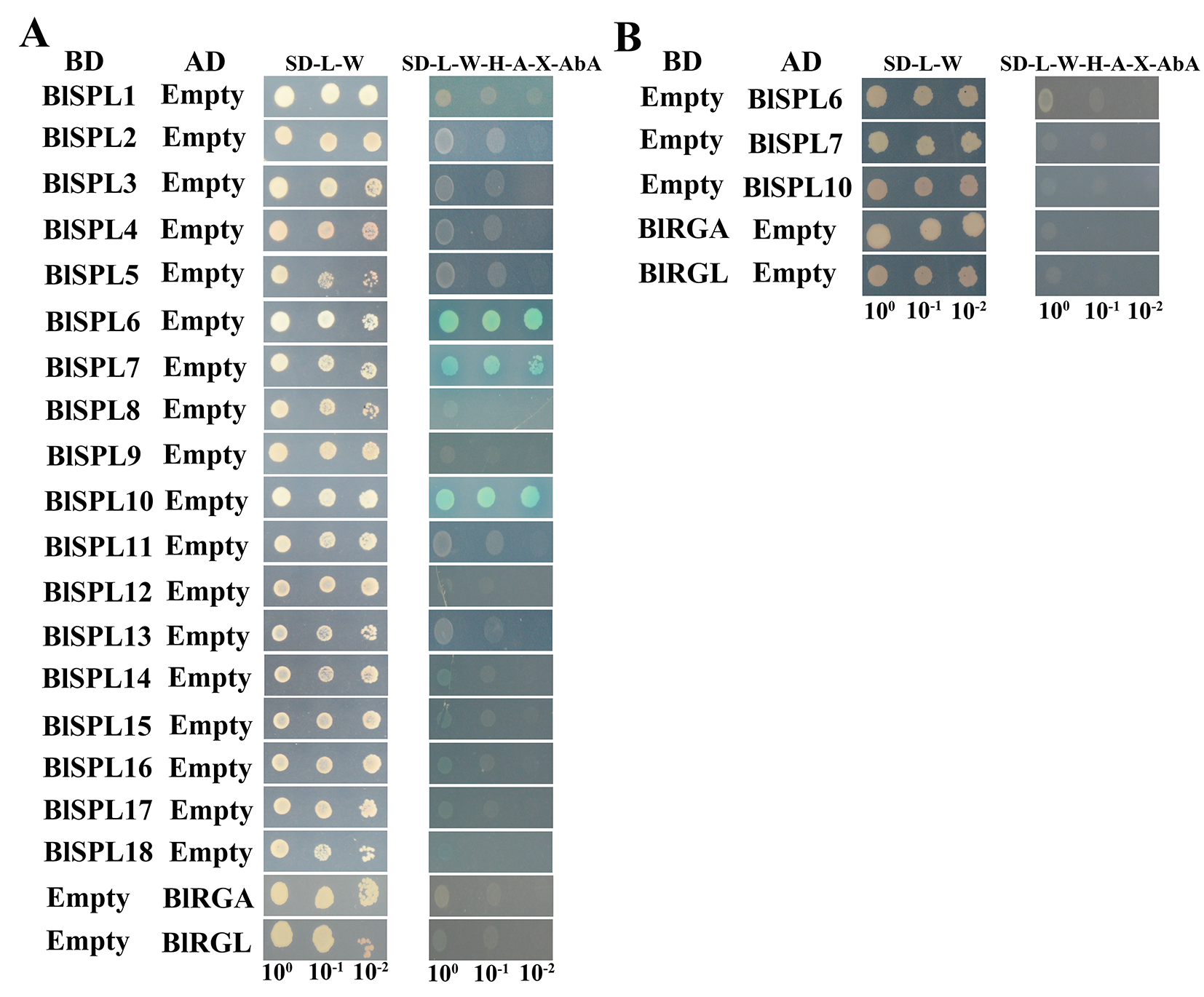

Supplement: Supplementary Figure S1 — Autoactivation and toxicity test for the BD fused constructs and AD fused constructs. (A) Autoactivation and toxicity of BD-BlSPLs, and AD-BlRGA and AD-BlRGL. (B) Autoactivation and toxicity of BD- BlRGA, BD-BlRGL, and AD-BlSPL6/7/10. BD, GAL4 binding domain; AD, GAL4 activation domain. Autoactivation was detected on the SD-Leu-Trp-His-Ade plates supplemented with 40 μg/ml X-α-gal and 125 ng/ml Aureobasidin A (SD-L-W-H-A-X-AbA). Toxicity was examined on the SD medium without leucine and tryptophan (SD-L-W). The yeast clones were grown with dilutions to 10−1 and 10−2. [file Image_1.TIF]

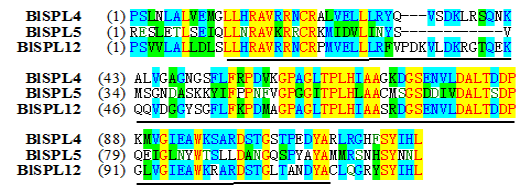

Supplement: Supplementary Figure S2 — Alignment of ANK/ANK-2 domain in BlSPL4, BlSPL5 and BlSPL10. The ANK/ANK-2 domain is marked by solid lines. [file Image_2.TIF]

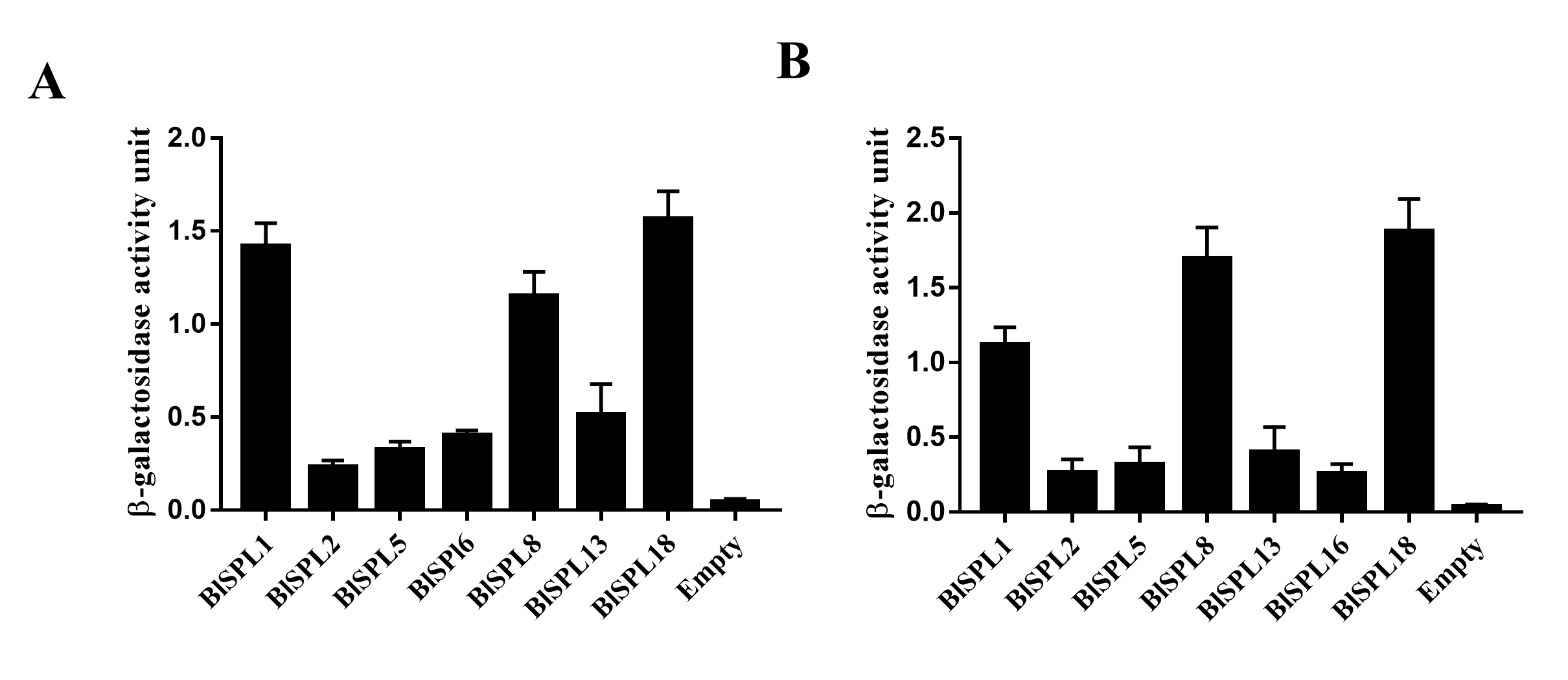

Supplement: Supplementary Figure S3 — The interaction strengths were determined through β-galactosidase activity assay. (A) The interaction strengths between BlSPLs and BlRGA. (B) The interaction strengths between BlSPLs and BlRGL. Empty, BD and AD vector without target genes, as a negative control. [file Image_3.TIF]

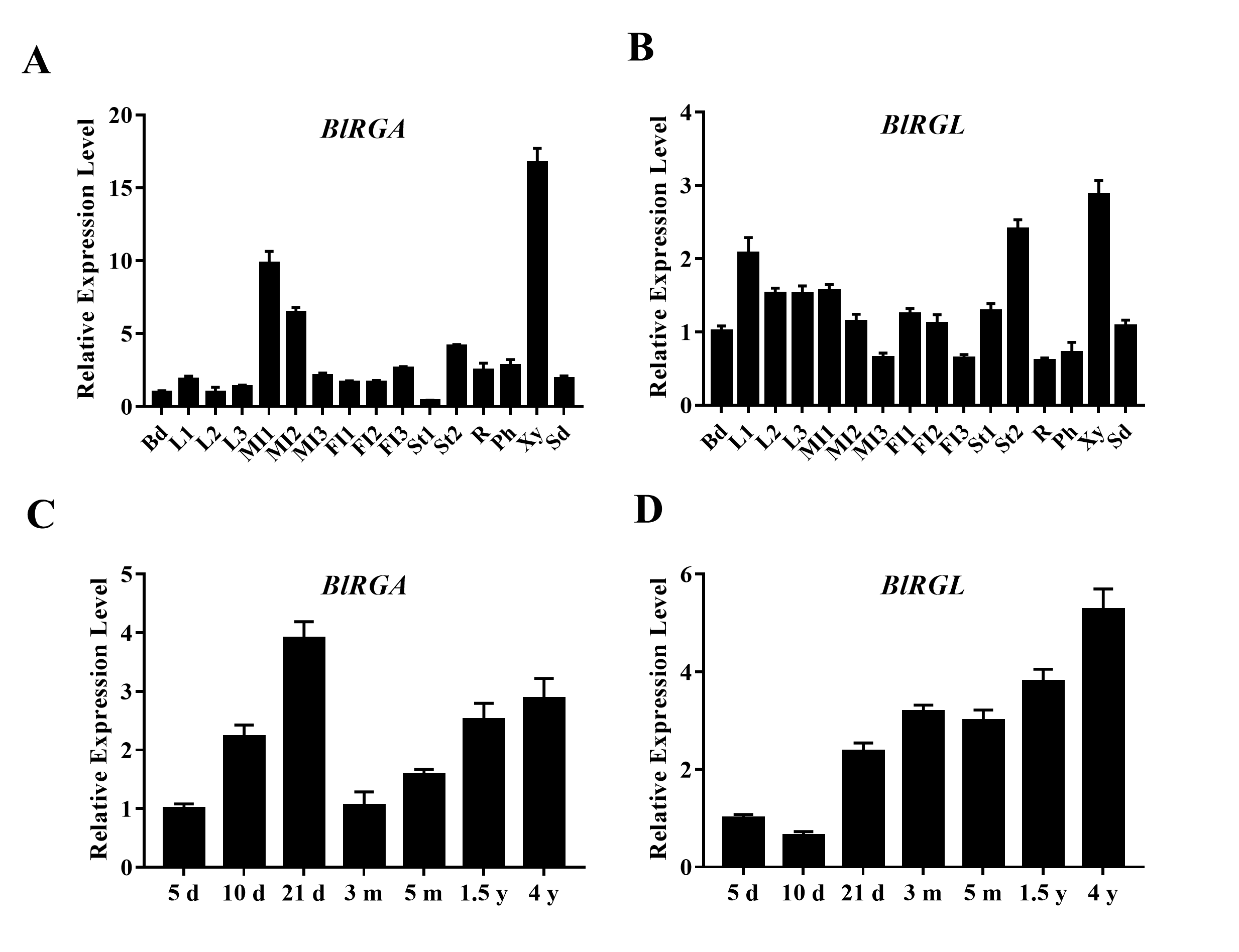

Supplement: Supplementary Figure S4 — Expression profiles of BlRGA and BlRGL. (A) Expression patterns of BlRGA in different tissues/organs. (B) Expression patterns of BlRGL in different tissues/organs. (C) Expression patterns of BlRGA in plants with different ages. (D) Expression patterns of BlRGL in plants with different ages. The same samples as figure 7 and 8 were used for the expression profiling of BlRGA and BlRGL. Transcript levels in buds and 5-day-old (5 d) seedlings were arbitrarily set to 1 and the levels in other tissues were given relative to this. Error bars represent standard deviations of mean value from three biological replicates and four technical replicates. [file Image_4.TIF]
