# Supplementary material for: Molecular Characterization of SQUAMOSA PROMOTER BINDING PROTEIN-LIKE (SPL) Gene Family in Betula luminifera
Source: Front Plant Sci. 2018 May 4;9:608. doi: 10.3389/fpls.2018.00608 (PMC5945835; doi:10.3389/fpls.2018.00608)
Supplement: Supplementary Table S7 — General information of SPL genes used for phylogenetic analysis. [file Table_7.DOC]

**Supplementary Table S7** General information of SPL genes selected for phylogenetic analysis.

| Gene | Accession no. or locus ID | Protein sequence of SBP-domain |
| --- | --- | --- |
| BlSPL1 | AGC92796 | CLVDGCTSDLSNCRDYHRRHKVCELHSKTPEVTICGQKQRFCQQCSRFHSLEEFDEGKRSCRKRLDGHNRRRRKPQ |
| BlSPL2 | KY548818 | CQAEGCNADLTHAKHYHRRHKVCEFHSKASTVVAAGLTQRFCQQCSRFHLLSEFDNGKRSCRKRLADHNRRRRKTH |
| BlSPL3 | KY548819 | CQVENCNADLTDAKHYHRRHKVCESHAKAPIAYVAGGQKRFCQQCSRFHDLSEFDEYKKSCRKRLAGHNERRRKSS |
| BlSPL4 | KY548820 | CQVDHCGADLSNAKDYHRRHKVCEMHSKASKALVGNVMQRFCQQCSRFHVLQEFDEGKRSCRRRLAGHNKRRRKTN |
| BlSPL5 | KY548821 | CQVDNCKEDLSNAKDYHRRHKVCEVHSKSGKALVANQMQRFCQQCSRFHPLSEFDEGKRSCRRRLAGHNRRRRKTQ |
| BlSPL6 | KY548822 | CQVEGCNLDLSSAKDYHRKHRVCESHSKSPKVTVGGRERRFCQQCSRFHGLSEFDEKKRSCRRRLSDHNARRRKPQ |
| BlSPL7 | KY548823 | CQVEGCNLDLKSAKDYHRRHRICECHSKSPKVIVAGMERRFCQQCSRFHELSEFDDKKRSCRRRLSDHNARRRRPQ |
| BlSPL8 | KY548824 | CQVEGCKLDLSDAKAYYSRHKVCGMHSKSPKVIVAGLEQRFCQQCSRFHQLPEFDQGKRSCRRRLAGHNERRRKPP |
| BlSPL9 | KY548825 | CLVDGCKTDLNICREYHRRHRVCERHSKTPVVIVGGKEQRFCQQCSRFHSLGEFDEAKRSCRKRLDGHNMRRRKSQ |
| BlSPL10 | KY548826 | CQVPGCEADISELKGYHRRHRVCLRCAHATAVVLEGETKRYCQQCGKFHVISDFDEGKRSCRRKLERHNNRRRRKP |
| BlSPL11 | KY548827 | CQVHGCNKDLSSSKDYHKRHKVCDVHSKTAKVVVNGIEQRFCQQCSRFHLLAEFDDGKRSCRKRLAGHNERRRKPQ |
| BlSPL12 | KY548828 | CQVEDCMADLSNAKDYHRRHKVCDMHSKASKALVGNVLQRFCQQCSRFHVLQEFDEGKRSCRRRLAGHNRRRRKTH |
| BlSPL13 | KY548829 | CQVYGCNKDLNASKDYHKRHKVCEVHSKTAKVIVNGIEQRFCQQCSRFHLLAEFDDGKRSCRKRLAGHNERRRKPQ |
| BlSPL14 | KY548830 | CQADECGVELQMAKAYHKRHKVCERHAKAAVVLVSGLRQRFCQQCSKFHEISQFDDNKKSCREKLAGHNERRRKTH |
| BlSPL15 | KY548831 | CQAEKCGADLTDAKRYHRRHKVCELHSKAPAVLVAGQRQRFCQQCSRFHELSEFDEAKRSCRRRLAGHNERRRKVG |
| BlSPL16 | KY548832 | CQVEGCHVPLLNAKDYHRRHKVCEMHSKAPRVVVLGLEQRFCQQCSRFHAVSEFDDSKRSCRRRLAGHNERRRKSA |
| BlSPL17 | KY548833 | CQAEKCTADLSDGKQYHKRHKVCEYHAKAQVVLVGGMRQRFCQQCSRFHELSEFDETKRSCRRRLAGHNERRRKNT |
| BlSPL18 | KY548834 | CLVDGCTSDLSKCRDYHRRHKVCEDHSKTPKVTIRGQEQRFCQQCSRFHSLGEFDEGKRSCRKRLDGHNRRRRKPQ |
| PtSPL1 | Potri.010G154000 | CQVEDCRADLSNAKDYHRRHKVCDVHSKASMALVGNVMQRFCQQCSRFHVLQEFDEGKRSCRRRLAGHNKRRRKTH |
| PtSPL2 | Potri.002G002400 | CQVDNCKEDLSKAKDYHRRHKVCQVHSKATKALVGKQMQRFCQQCSRFHPLTEFDEGKRSCRRRLAGHNRRRRKTQ |
| PtSPL3 | Potri.010G026200 | CQVPSCEADISELKGYHRRHRVCLGCANATAVVLDGETKRYCQQCGKFHVLSDFDEGKRSCRRKLERHNNRRRRKP |
| PtSPL4 | Potri.008G197000 | CQVPGCETDISELKGYHRRHKVCLRCATATAVVLDEQTKRYCQQCGKFHVLSDFDEGKRSCRRKLERHNNRRRRKP |
| PtSPL5 | Potri.008G098600 | CQVEDCRADLSNAKDYHRRHKVCNAHSKASKALVGNVMQRFCQQCSRFHVLQEFDEGKRSCRRRLAGHNKRRRKTH |
| PtSPL6 | Potri.014G114300 | CQVEDCGVDLSNAKDYHRRHKVCEMHSKASKALVGNAMQRFCQQCSRFHVLQEFDEGKRSCRRRLAGHNKRRRKTN |
| PtSPL7 | Potri.002G188700 | CQVEDCGVDLSNAKDYHRRHKVCEMHSKASKALVGNVMQRFCQQCSRFHVLQEFDEGKRSCRRRLAGHNKRRRKTN |
| PtSPL8 | Potri.002G142400 | CQVEGCHVALVNAKGYHRRHKVCEMHSKAAKVIVLGLEQRFCQQCSRFHVVSEFDDAKRSCRRRLAGHNERRRKGS |
| PtSPL9 | Potri.005G258700 | CQVDNCKENLTTAKDYHRRHKVCEVHSKATKALVGKQMQRFCQQCSRFHPLTEFDEGKRSCRRRLAGHNRRRRKTQ |
| PtSPL11 | Potri.003G172600^b^ | CQVEGCNLDLSSAKDYHRKHRVCESHSKCPKVIVAGLERRFCQQCSRFHGLSEFDEKKKSCRRRLSDHNARRRKQP |
| PtSPL12 | Potri.008G097900 | CQVYGCNKDLSSSKDYHKRHKVCEVHSKTPQVIVDGNEQRFCQQCSRFHLLVDFDDGKRSCRKRLAGHNERRRKPQ |
| PtSPL13 | Potri.010G154300 | CQVYDCNKDLSSSKDYHKRHKVCEVHTKTPQVIVNGNEQRFCQQCSRFHLLVEFDDGKRSCRKRLAGHNERRRKPQ |
| PtSPL14 | Potri.015G098900 | CLVDGCNSDLSACRDYHRRHKVCELHSKTPQVTVGGQKQRFCQQCSRFHSLEEFDEGKRSCRKRLDGHNRRRRKPQ |
| PtSPL15 | Potri.012G100700 | CLVDGCNSDLSTCRDYHRRHKVCELHSKTPQVTIGGQKQRFCQQCSRFHSLEEFDEGKRSCRKRLDGHNRRRRKPQ |
| PtSPL16 | Potri.011G055900 | CQADNCTSDLADAKRYHRRHKVCEFHAKAPFAPVNGLQQRFCQQCSRFHDLSEFDDSKRSCRRRLAGHNERRRKSS |
| PtSPL17 | Potri.016G048500^c^ | CQVEGCKVDLSDAKTYYSRHKVCSMHSKSPRVIVAGLVQRFCQQCSRFHLLPEFDQGKRSCRRRLAGHNERRRKPP |
| PtSPL18 | Potri.001G058600 | CLVDGCTSDLSKCRDYHRRHKVCEFHSKSSQVFIKGQEQRFCQQCSRFHSLGEFDEGKRSCRKRLDGHNRRRRKPQ |
| PtSPL19 | Potri.001G055900 | CQVEGCNLDLSSAKDYHRKHRVCESHSKCQKVIVAGLERRFCQQCSRFHGLSEFDEKKKSCRRRLSDHNARRRKQP |
| PtSPL20 | Potri.001G398200 | CQVEKCGANLTDAKRYHRRHKVCEVHAKSPAVVVAGLRQRFCQQCSRFHELVEFDETKRSCRRRLAGHNERRRKST |
| PtSPL21 | Potri.002G142200 | CQAEGCNADLTHAKHYHRRHKVCEFHSKASTVIAAGLTQRFCQQCSRFHILSEFDNGKRSCRKRLADHNRRRRKSH |
| PtSPL22 | Potri.003G169400 | CLVDGCTSDLTKCRDYHRRHKVCELHSKSRQVFIKGQEQRFCQQCSRFHSLGEFDEGKRSCRKRLDGHNRRRRKSQ |
| PtSPL23 | Potri.004G046700 | CQVKNCTTDMTDAKRYHKRHKVCEFHAKASSVLVNGVEQRFCQQCSRFHDLSEFDDSKRSCRRRLAGHNERRRKSS |
| PtSPL24 | Potri.007G138800 | CQVEKCTANLTDAKQYHRRHKVCGHHAKAQVVLVAGIRQRFCQQCSRFHELSEFDETKRSCRRRLAGHNERRRKNV |
| PtSPL25 | Potri.011G116800^d^ | CQVEKCTANLTDAKQYHRRHKVCGHHAKAQVVLVAGIRQRFCQQCSRFHELSEFDETKRSCRRRLAGHNERRRKNV |
| PtSPL26 | Potri.014G057700 | CQAEGCNADLTHAKHYHRRHKVCEFHSKASTVIAAGLTQRFCQQCSRFHLLSEFDNGKRSCRKRLADHNRRRRKSH |
| PtSPL27 | Potri.014G057800 | CQVEGCHVALLNAKDYHRRHKVCEMHSKAPKVIVLGLEQRFCQQCSRFHVVSEFDDAKRSCRRRLAGHNERRRKSS |
| PtSPL28 | Potri.015G060400 | CQVYDCNKDLSSSKEYHKRHKVCEVHSRTAKVIVNGIEQRFCQQCSRFHLLAEFDDGKRSCRKRLAGHNERRRKPQ |
| PtSPL29 | Potri.018G149900 | CQVEGCNLDLKSAKDYHRRHRICEKHSKSPKVIVAGMERRFCQQCSRFHELSEFDDKKRSCRRRLSDHNARRRRPQ |
| OsSPL1 | LOC_Os01g18850.1 | CQVDGCTVNLSSARDYNKRHKVCEVHTKSGVVRIKNVEHRFCQQCSRFHFLQEFDEGKKSCRSRLAQHNRRRRKVQ |
| OsSPL2 | LOC_Os01g69830.1 | CSVEGCAADLSKCRDYHRRHKVCEAHSKTAVVTVAGQQQRFCQQCSRFHLLGEFDEEKRSCRKRLDGHNKRRRKPQ |
| OsSPL3 | LOC_Os02g04680.1 | CQVEGCNVDLSSAKPYHRKHRVCEPHSKTLKVIVAGLERRFCQQCSRFHGLAEFDQKKRSCRRRLHDHNARRRKPQ |
| OsSPL4 | LOC_Os02g07780.1 | CQVEGCGVELVGVKDYHRKHRVCEAHSKFPRVVVAGQERRFCQQCSRFHALSEFDQKKRSCRRRLYDHNARRRKPQ |
| OsSPL5 | LOC_Os02g08070.1 | CQAEGCKADLSAAKHYHRRHKVCDFHAKAAAVLAAGKQQRFCQQCSRFHVLAEFDEAKRSCRKRLTEHNRRRRKPT |
| OsSPL6 | LOC_Os03g61760.1 | CQVEGCTADLTGVRDYHRRHKVCEMHAKATTAVVGNTVQRFCQQCSRFHPLQEFDEGKRSCRRRLAGHNRRRRKTR |
| OsSPL7 | LOC_Os04g46580.1 | CQVEGCDITLQGVKEYHRRHKVCEVHAKAPRVVVHGTEQRFCQQCSRFHVLAEFDDAKKSCRRRLAGHNERRRRSN |
| OsSPL8 | LOC_Os04g56170.1 | CQAEGCKADLSSAKRYHRRHKVCEHHSKAPVVVTAGLHQRFCQQCSRFHLLDEFDDAKKSCRKRLADHNRRRRKSK |
| OsSPL9 | LOC_Os05g33810.1 | CQVPGCEADIRELKGYHRRHRVCLRCAHAAAVMLDGVQKRYCQQCGKFHILLDFDEDKRSCRRKLERHNRRRRKPD |
| OsSPL10 | LOC_Os06g44860.1 | CQAEGCKADLSGAKHYHRRHKVCEYHAKASVVAASGKQQRFCQQCSRFHVLTEFDEAKRSCRKRLAEHNRRRRKPA |
| OsSPL11 | LOC_Os06g45310.1 | CQVEGCGLELGGYKEYYRKHRVCEPHTKCLRVVVAGQDRRFCQQCSRFHAPSEFDQEKRSCRRRLSDHNARRRKPQ |
| OsSPL12 | LOC_Os06g49010.1 | CQVEGCKVDLSSAREYHRKHKVCEAHSKAPKVIVSGLERRFCQQCSRFHGLAEFDQKKKSCRRRLSDHNARRRKPQ |
| OsSPL13 | LOC_Os07g32170.1 | CQVERCGVDLSEAGRYNRRHKVCQTHSKEPVVLVAGLRQRFCQQCSRFHELTEFDDAKRSCRRRLAGHNERRRKSA |
| OsSPL14 | LOC_Os08g39890.1 | CQVEGCGADLSGIKNYYCRHKVCFMHSKAPRVVVAGLEQRFCQQCSRFHLLPEFDQGKRSCRRRLAGHNERRRRPQ |
| OsSPL15 | LOC_Os08g40260.1 | CQVDDCRADLTNAKDYHRRHKVCEIHGKTTKALVGNQMQRFCQQCSRFHPLSEFDEGKRSCRRRLAGHNRRRRKTQ |
| OsSPL16 | LOC_Os08g41940.1 | CAVDGCKEDLSKCRDYHRRHKVCEAHSKTPLVVVSGREMRFCQQCSRFHLLQEFDEAKRSCRKRLDGHNRRRRKPQ |
| OsSPL17 | LOC_Os09g31438.1 | CQVEGCGVDLSGVKPYYCRHKVCYMHAKEPIVVVAGLEQRFCQQCSRFHQLPEFDQEKKSCRRRLAGHNERRRKPT |
| OsSPL18 | LOC_Os09g32944.1 | CAVDGCKADLSKHRDYHRRHKVCEPHSKTPVVVVSGREMRFCQQCSRFHLLGEFDEAKRSCRKRLDGHNRRRRKPQ |
| OsSPL19 | LOC_Os11g30370.1 | CSVDGCRSDLSRCRDYHRRHKVCEAHAKTPVVVVAGQEQRFCQQCSRFHNLAEFDDGKKSCRKRLDGHNRRRRKPQ |
| AtSPL1 | At2g47070 | CQVENCEADLSKVKDYHRRHKVCEMHSKATSATVGGILQRFCQQCSRFHLLQEFDEGKRSCRRRLAGHNKRRRKTN |
| AtSPL2 | At5g43270 | CQVEGCNLDLSSAKDYHRKHRICENHSKFPKVVVSGVERRFCQQCSRFHCLSEFDEKKRSCRRRLSDHNARRRKPN |
| AtSPL3 | At2g33810 | CQVESCTADMSKAKQYHKRHKVCQFHAKAPHVRISGLHQRFCQQCSRFHALSEFDEAKRSCRRRLAGHNERRRKST |
| AtSPL4 | At1g53160 | CQVDRCTADMKEAKLYHRRHKVCEVHAKASSVFLSGLNQRFCQQCSRFHDLQEFDEAKRSCRRRLAGHNERRRKSS |
| AtSPL5 | At3g15270 | CQVDRCTVNLTEAKQYYRRHRVCEVHAKASAATVAGVRQRFCQQCSRFHELPEFDEAKRSCRRRLAGHNERRRKIS |
| AtSPL6 | At1g69170 | CQVYGCSKDLSSSKDYHKRHRVCEAHSKTSVVIVNGLEQRFCQQCSRFHFLSEFDDGKRSCRRRLAGHNERRRKPA |
| AtSPL7 | At5g18830 | CQVPDCEADISELKGYHKRHRVCLRCATASFVVLDGENKRYCQQCGKFHLLPDFDEGKRSCRRKLERHNNRRKRKP |
| AtSPL8 | At1g02065 | CQAEGCNADLSHAKHYHRRHKVCEFHSKASTVVAAGLSQRFCQQCSRFHLLSEFDNGKRSCRKRLADHNRRRRKCH |
| AtSPL9 | At2g42200 | CQVEGCGMDLTNAKGYYSRHRVCGVHSKTPKVTVAGIEQRFCQQCSRFHQLPEFDLEKRSCRRRLAGHNERRRKPQ |
| AtSPL10 | At1g27370 | CQIDGCELDLSSSKDYHRKHRVCETHSKCPKVVVSGLERRFCQQCSRFHAVSEFDEKKRSCRKRLSHHNARRRKPQ |
| AtSPL11 | At1g27360 | CQIDGCELDLSSAKGYHRKHKVCEKHSKCPKVSVSGLERRFCQQCSRFHAVSEFDEKKRSCRKRLSHHNARRRKPQ |
| AtSPL12 | At3g60030 | CQVDNCGADLSKVKDYHRRHKVCEIHSKATTALVGGIMQRFCQQCSRFHVLEEFDEGKRSCRRRLAGHNKRRRKAN |
| AtSPL13 | At5g50570 | CLVDGCDSDFSNCREYHKRHKVCDVHSKTPVVTINGHKQRFCQQCSRFHALEEFDEGKRSCRKRLDGHNRRRRKPQ |
| AtSPL14 | At1g20980 | CQVDNCTEDLSHAKDYHRRHKVCEVHSKATKALVGKQMQRFCQQCSRFHLLSEFDEGKRSCRRRLAGHNRRRRKTT |
| AtSPL15 | At3g57920 | CQVEGCRMDLSNVKAYYSRHKVCCIHSKSSKVIVSGLHQRFCQQCSRFHQLSEFDLEKRSCRRRLACHNERRRKPQ |
| AtSPL17 | At5g50670 | CLVDGCDSDFSNCREYHKRHKVCDVHSKTPVVTINGHKQRFCQQCSRFHALEEFDEGKRSCRKRLDGHNRRRRKPQ |

**Supplementary Table S8** Data sources of SPL genes used for phylogenetic analysis.

| Species | Gene symbol | Data source |
| --- | --- | --- |
| *Betula luminifera* | BlSPL | NCBI |
| *Arabidopsis thaliana* | AtSPL | http://www.arabidopsis.org/ |
| *Oryza sativa* | OsSPL | http://rice.plantbiology.msu.edu |
| *Populus trichocarpa* | PtSPL | Li et al(Li and Lu, 2014) |

**References:**

**Li C, Lu S** (2014) Molecular characterization of the SPL gene family in Populus trichocarpa. BMC Plant Biol **14:** 131
