# Supplementary material for: Molecular Characterization of SQUAMOSA PROMOTER BINDING PROTEIN-LIKE (SPL) Gene Family in Betula luminifera
Source: Front Plant Sci. 2018 May 4;9:608. doi: 10.3389/fpls.2018.00608 (PMC5945835; doi:10.3389/fpls.2018.00608)
Supplement: Supplementary Table S8 — Data sources of SPL genes used for phylogenetic analysis. [file Table_8.doc]

**Supplementary Table S8** Data sources of SPL genes used for phylogenetic analysis.

| Species | Gene symbol | Data source |
| --- | --- | --- |
| *Betula luminifera* | BlSPL | NCBI |
| *Arabidopsis thaliana* | AtSPL | http://www.arabidopsis.org/ |
| *Oryza sativa* | OsSPL | http://rice.plantbiology.msu.edu |
| *Populus trichocarpa* | PtSPL | Li et al(Li and Lu, 2014) |

**References:**

**Li, C., and Lu, S.** (2014). Molecular characterization of the SPL gene family in Populus trichocarpa. BMC PLANT BIOL **14,** 131.
